# Coronary Artery Blood Flow Imaging Using 3D Flow MRI

**DOI:** 10.1002/mrm.70147

**Published:** 2025-10-22

**Authors:** Denise Lichthardt, Michaela Schmidt, Jens Wetzl, Peter Speier, Manuel Hein, Christopher Schuppert, Armin M. Nagel, Daniel Giese

**Affiliations:** ^1^ Institute of Radiology, University Hospital Erlangen, Friedrich‐Alexander‐Universität Erlangen‐Nürnberg (FAU) Erlangen Germany; ^2^ Research & Clinical Translation, Magnetic Resonance Siemens Healthineers AG Erlangen Germany; ^3^ Department of Cardiology and Angiology Medical Center – University of Freiburg, Faculty of Medicine, University of Freiburg Freiburg Germany; ^4^ Department of Diagnostic and Interventional Radiology Medical Center – University of Freiburg, Faculty of Medicine, University of Freiburg Freiburg Germany

**Keywords:** cardiovascular magnetic resonance angiography, coronary artery disease, coronary imaging, flow imaging, phase contrast MRI

## Abstract

**Purpose:**

In this work, a 3D phase contrast (PC) technique is presented, which enables flow velocity measurements in three spatial directions, simultaneously covering the proximal left (LCA) and right (RCA) coronary arteries and yielding both morphological and flow information.

**Methods:**

Data is acquired in a single diastolic cardiac phase during free breathing using an isotropic (Δ*x* = 1.2 mm) 3D PC sequence with respiratory gating and compressed sensing acceleration (factor 14). The reference and flow‐encoded datasets are obtained from separate scans instead of one interleaved measurement, enabling inter‐scan motion correction through image co‐registration. The proposed method is validated in a flow phantom and its feasibility is demonstrated in 16 volunteers (8 female, 59.3 ± 16.5 years) and 3 patients (1 female, 71.0 ± 9.6 years) with known CAD, where additional CTA and CT‐FFR data were obtained. Data were acquired on a 3 T MRI system and post‐processed using in‐house developed software.

**Results:**

Flow phantom experiments demonstrated good agreement between the proposed method and a typical 4D Flow acquisition. Mean average velocities in the proximal coronary arteries of volunteers were found to be 11.9 ± 3.3 cm s^−1^ (LCA) and 8.4 ± 2.6 cm s^−1^ (RCA). For patients, increases in velocities correlated with the locations of stenoses and reduced CT‐FFR values.

**Conclusion:**

The feasibility of 3D‐PC‐MRI for non‐invasive 3D blood flow velocity imaging in the coronary arteries is demonstrated. Simultaneous acquisitions of high‐resolution morphology and velocities could offer new opportunities for diagnosing and monitoring CAD.

## 1 Introduction

4D Flow MRI offers the unique possibility to measure time‐resolved velocities in 3D using phase contrast MRI [1] and is typically applied for the assessment of flow within the heart and large vessels. Its applicability in the coronary arteries (CA), however, remains extremely challenging due to breathing‐ and heartbeat‐induced motion, the small vessel diameters, and limited signal‐to‐noise ratios [2]. Although 2D phase contrast MRI (2D Flow) has been shown to be feasible and can be used to quantify flow in the CA, its use often remains limited as flow is quantified through a single plane only [3, 4, 5]. Furthermore, the planning of 2D Flow acquisitions in the CA is complicated by cardiac and respiratory motion, small vessel sizes, and vessel tortuousness. On the other hand, 3D morphological assessment of CA using MRI, denoted as coronary magnetic resonance angiography (CMRA), has advanced to a robust technique and shown promising results when validated against computed tomography angiography (CTA) and invasive coronary angiography during percutaneous coronary interventions (PCI) [6, 7]. For hemodynamic measurements in the CA, the assessment of the fractional flow reserve (FFR) during PCI is the clinical gold standard [8]. Recent advances have shown high diagnostic accuracy of the calculation of a virtual‐FFR value based on morphological CTA (CT‐FFR) [9]. Being non‐invasive, CTA‐based methods provide greater patient comfort compared to PCI; however, they rely on retrospective calculations of hemodynamics instead of directly measuring flow information.

In this work, we propose a novel MRI technique to measure 3D blood flow velocities simultaneously in the proximal left and right CA (LCA and RCA) during free‐breathing without the use of a contrast agent, following up on initial work on this topic [10, 11, 12]. The acquisition combines methods developed for CMRA and 4D Flow. To mitigate cardiac motion, the measurement is performed during a single cardiac phase in diastole. During this phase, the heart remains comparably quiescent in patients with sinus rhythm and at rest, and peak flow velocities in the CA are higher [13, 14]. Measuring velocities during diastole therefore not only reduces cardiac motion effects compared to systole but is also expected to improve velocity quantification and identification of flow abnormalities. Breathing motion is compensated by synchronizing the measurement with end‐expiration and additionally applying a retrospective motion correction. A tolerable scan time is achieved by using high undersampling factors in combination with a dedicated compressed sensing (CS) reconstruction [15]. Artifacts due to flow displacement [16] are further reduced by a retrospective correction. For post‐processing, a tool for 3D visualization and quantitative evaluation was developed.

The velocity measurements in the LCA and RCA were validated using 4D Flow in a realistic phantom setup, and reference values were acquired in 16 volunteers without known coronary artery disease (CAD). Intra‐session scan‐rescan analysis was performed in 12 of these volunteers. The technique's feasibility to measure flow velocity abnormalities was finally demonstrated in 3 patients with confirmed CAD by comparing results to CTA and CT‐FFR.

## 2 Methods

### 2.1 MRI Sequence and Reconstruction

Measurements were performed on 3 T MRI systems (MAGNETOM Skyra and MAGNETOM Vida, Siemens Healthineers, Forchheim, Germany) using an RF spoiled gradient echo research sequence with phase‐contrast flow encoding in all three dimensions, referred to as 3D‐PC‐MRI in the following. Adiabatic *T*_2_ preparation pulses [17] and the spectral attenuated inversion recovery technique [18] were used to improve the image contrast and suppress fat signal. Saturation bands were placed on the arms to avoid aliasing artifacts. A pseudo‐random sampling pattern with a nominal acceleration factor of 14 was used for all four flow encoding acquisitions. Detailed sequence parameters are given in Table 1.

**TABLE 1:** Sequence parameters for in vivo measurements.

TE	3.95 ms
TR	5.98 ms
Flip angle	15°
VENC	50 cm s^−1^
CS undersampling factor	14
Resolution (Δ*x*)^3^	(1.2 mm)^3^
Total acquisition time (TA)	16:07 ± 4:47 min
Navigator acceptance window	±4.00 mm

For in vivo measurements, navigator‐based gating was employed to reduce motion artifacts induced by respiration [19]. Previous studies observed that periods with the least motion of the CA occur at end systole and mid diastole [13, 20] and peak flow rates were found in diastole [14, 21]. Hence, measurements were performed in diastole only, using an acquisition window size of approximately 120 ms per cardiac cycle in which 20 k‐space lines were acquired. The optimum trigger delay for each subject was determined based on a preceding time‐resolved 2D cine scan acquired in axial orientation to capture the quiescent period of the right coronary artery segment located a few centimeters distal to the ostium.

**FIGURE 1:**
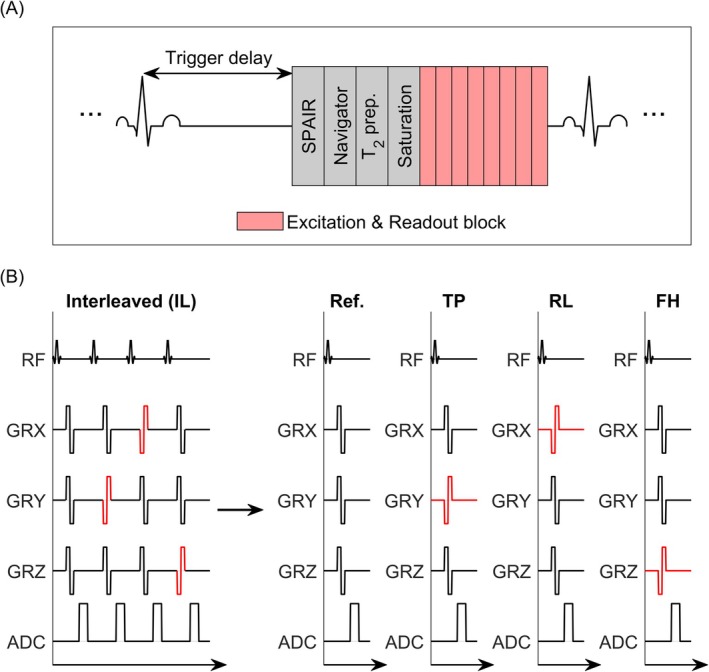
Data acquisition sequence for the proposed 3D phase contrast (PC) method. (A) Schematic pulse sequence. After the trigger delay following the detection of the R‐wave, a spectral attenuated inversion recovery (SPAIR) pulse is played out for fat suppression, followed by respiratory navigators, T2 preparation pulses, and signal saturation pulses placed on the arms. Subsequently, a series of 20 k‐space lines are acquired (only eight are shown). (B) Sequence modifications based on an interleaved PC sequence. Instead of one interleaved acquisition, the measurement is split into four separate scans: one for reference, through‐plane (TP) flow‐encoded, right–left (RL) flow‐encoded, and foot‐head (FH) flow‐encoded datasets respectively. For simplicity, only flow compensating and flow encoding (red) gradients are shown.

For flow encoding, a four‐point symmetric encoding scheme was used, resulting in the acquisition of four datasets, differing in their flow‐encoding gradients [22]. Although the datasets are commonly acquired in an interleaved fashion, the proposed technique splits the acquisition into four consecutive scans, each of which has a respective scan time of approximately 25% of the time required for an interleaved sequence. This approach enables the separation of motion effects into intra‐ and inter‐scan motion, the latter of which can be corrected by co‐registration. Compared to interleaved scanning, the effect of intra‐scan motion is thereby reduced to one‐fourth of the total scan time. Furthermore, a repetition of a motion‐corrupted flow encoding scan is possible without the need for reacquiring all four datasets. The sequence is illustrated in Figure 1.

Reconstruction was performed on‐line on a GPU and consisted of a compressed sensing optimization with data consistency and wavelet regularization [15]. A single set of coil sensitivity maps was calculated applying ESPIRIT on the first flow encoded acquisition [23]. Each flow encoding acquisition was reconstructed separately, followed by an image‐based registration, a phase‐contrast calculation, and a concomitant field correction [24].

### 2.2 Phantom Experiments

**FIGURE 2:**
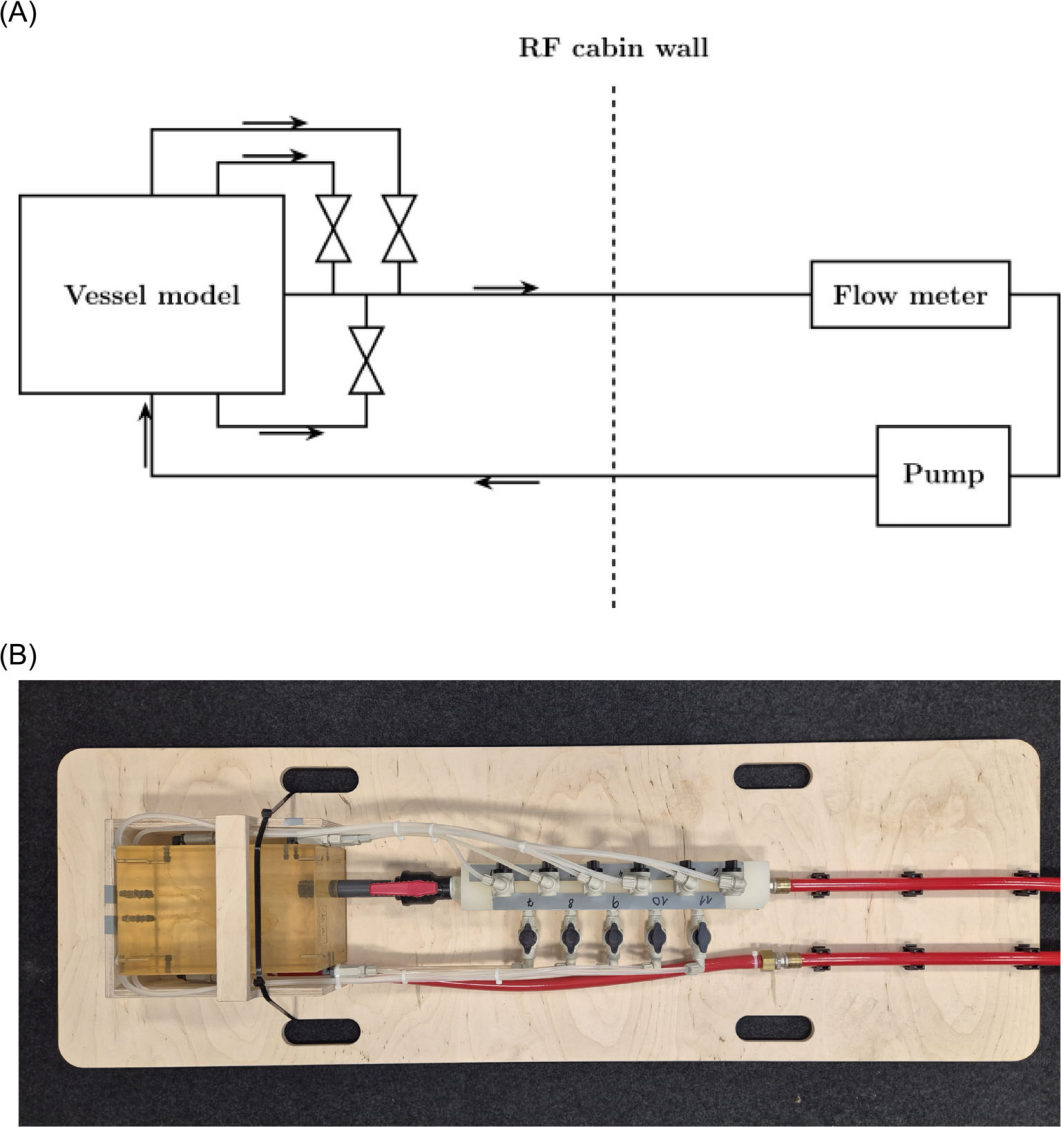
Flow phantom setup. (A) A schematic illustration of a realistic flow phantom, where the flow directions are indicated by arrows. The setup consisted of two parts. The part outside the radiofrequency (RF) cabin consisted of a pump, a flowmeter, and an electromechanical valve. The part inside the scanner consisted of a vessel model with one inlet and 11 individually adaptable outlets leading back to the main tube. (B) Photograph of the realistic flow phantom.

For validation, measurements were performed on a flow phantom, eliminating the influence of physiologic variations, breathing motion, and enabling time‐resolved 4D Flow acquisitions. The phantom consisted of a model of the aortic arch, the great vessels, and the CA (Ref: T‐R‐N‐002, Elastrat Sàrl, Geneva, Switzerland). The ascending aorta of the vessel model is shaped elliptically with diameters of 28 × 30 mm. The aortic arch and descending aorta have diameters of 26.0 and 20.0 mm, respectively. The right coronary artery has a diameter of 4.0 mm in the proximal branch, which bifurcates into two vessels with diameters of 2.0 mm each. The left coronary artery has a diameter of 4.0 mm in the proximal section, decreasing to 3.5 mm in the mid‐section. The vessel model was connected to a pump (Stratos PICO Z 25/1‐6, Wilo SE, Dortmund, Germany). The flow rate was adjusted by changing the pump height and left constant during each experiment. Valves at each outlet of the model were used to either block or enable flow through the corresponding CA and can therefore be employed to mimic stenoses. The phantom setup is schematically illustrated in Figure 2A,B.

Measurements during open and closed outlet valves were performed to investigate the feasibility of detecting hemodynamic changes. Furthermore, a vendor‐provided 4D Flow sequence with a single time‐frame was used as a reference acquisition [1]. A GRAPPA acceleration factor of 2 in the phase‐encoding direction, no *T*_2_ preparation, and no fat suppression were used while other parameters were matched to the ones listed in Table 1. Paired two‐tailed *t*‐tests were performed to compare quantitative flow velocities obtained with the two methods. Each value pair corresponds to flow velocities acquired at the same slice location along the CA using 3D‐PC‐MRI and 4D flow. The sample size corresponds to the number of slices in the proximal coronary artery, allowing a maximum distance of 25 mm from the ostium.

To quantify background flow effects due to eddy currents, data was additionally acquired without flow by turning the pump off.

An additional phantom geometry (Figure S1) was used to further validate flow velocity quantification. For this setup, the vessel model was replaced by a tube with a cross‐sectional area of approximately 200 mm^2^ consisting of two parallel sections, connected by a curved section. The details of these experiments and results are presented in Supporting Information.

### 2.3 Study Population

In vivo measurements were performed on 16 healthy volunteers without known cardiovascular disease (8 female, 59.3 ± 16.5 years). To analyze reproducibility, 14 volunteers were rescanned within the same session under the same conditions, and one volunteer was rescanned on a different day. Since 3D‐PC‐MRI requires 3 flow encoded and 1 reference scan, a total of 31 × 4 scans were therewith acquired. In two volunteers, one set of 4 scans (6%) needed to be excluded from postprocessing due to insufficient image quality, such that 12 scan‐rescan pairs remained for intra‐session scan‐rescan analysis. In four volunteers, image quality was inferior for just one of the datasets, which was reacquired without having to repeat all four scans. Note that three of these volunteers were scanned in the early stages of this study when experience with this sequence and the achievable level of image quality was limited. Hence, rescans were not only acquired in cases of insufficient quality, but also in cases where the image quality of one dataset was deemed acceptable for post‐processing, but was inferior to that of the other datasets. In these cases, rescanning aimed to homogenize image quality across datasets; however, it did not always yield improvements. If not, the initial scan was used for this study rather than the rescan. Only in one case was the rescan used.

Four patients with known CAD were scanned using the proposed sequence. Based on clinically indicated CTA examinations, proximal stenoses in the LCX and RCA were identified in patient 1 (male, 59 years) in addition to a distal (not within the cover range of our proposed method) stenosis in the LAD. A partial obstruction of the proximal LM was confirmed for patient 2 (male, 74 years). Patient 3 (female, 82 years) was diagnosed with three‐vessel disease, accompanied by proximal stenoses in both the LM and the RCA. Data acquired on patient 4 (male, 69 years, stenoses in the RCA, LAD, LCX) had to be excluded from post‐processing due to poor image quality.

The study was approved by the local institutional review board. All experiments were performed in accordance with the relevant guidelines and regulations. Written informed consent was obtained from all participants prior to the measurements.

### 2.4 Data Correction

The effect of flow displacement was accounted for using the single‐step displacement correction algorithm developed by Thunberg et al. [25]. The implementation was based on Roberts et al. [26].

For all in vivo scans, inter‐scan registration was performed using Matlab R2021b (The MathWorks Inc., Natick, Massachusetts, USA). First, magnitude and phase images were cropped to a region of interest covering the whole heart. Then, a 3D edge filter using the approximate Canny algorithm [27] was applied to the magnitude images, yielding image contours. The contours of the three flow‐encoded datasets were then registered to those of the reference dataset using a 3‐parametric translational transformation. The resulting transformations were also applied to the corresponding cropped phase images prior to background phase correction. Subsequently, the magnitude images were averaged to improve the SNR of the anatomical image.

### 2.5 Data Analysis

**FIGURE 3:**
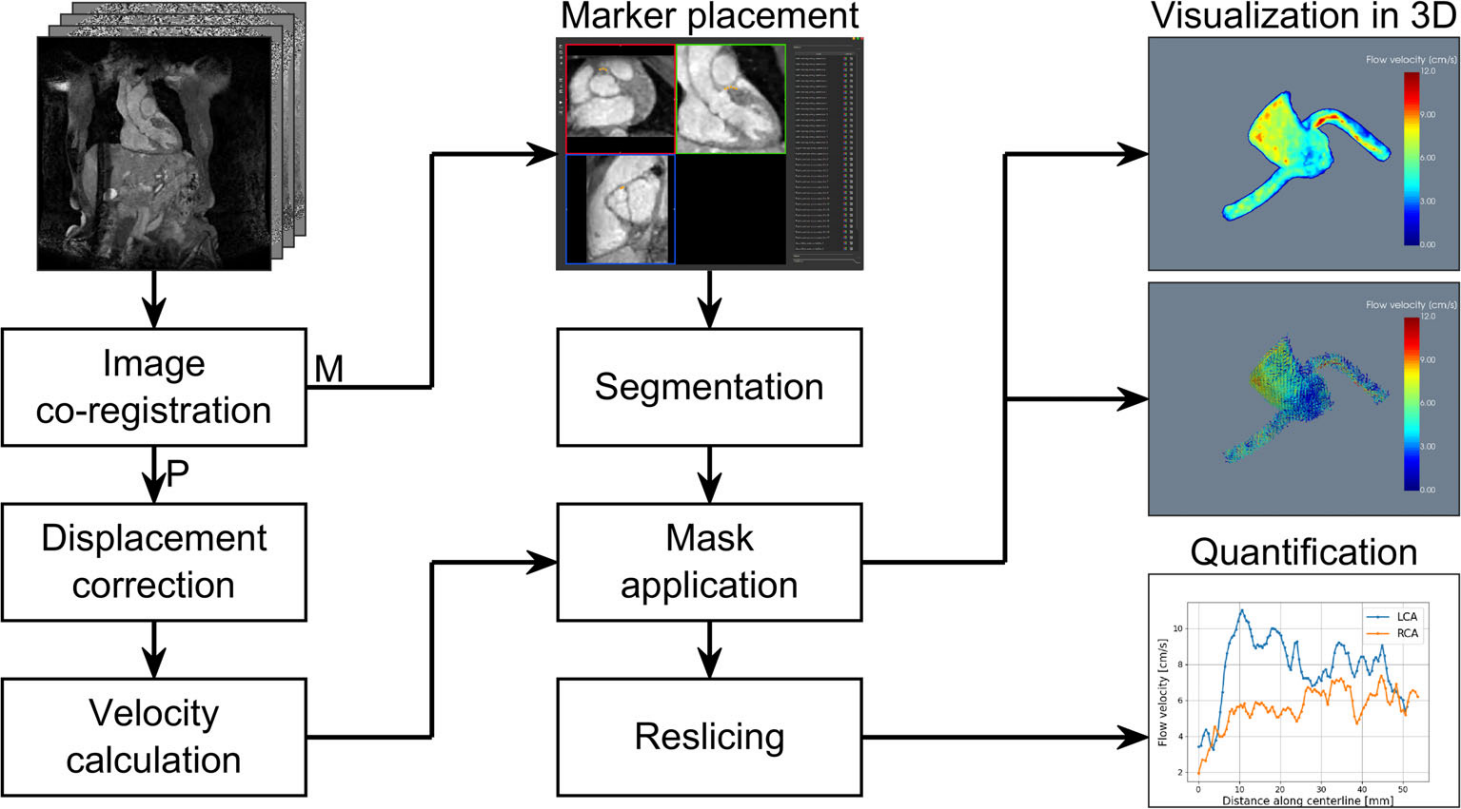
Postprocessing workflow. Both magnitude and phase images undergo co‐registration to account for inter‐scan motion. Subsequently, magnitude and phase images take separate paths through the postprocessing pipeline, marked by P and M, respectively. Phase images undergo spin displacement correction, followed by the calculation of a 3D velocity volume. Based on the magnitude images and manually set marker points along the vessel centerlines, a segmentation mask is calculated and applied to the velocity volume and visualized interactively using the 3D viewer. For quantification, the maximum velocity per slice perpendicular to the centerline is plotted against the distance along the centerline from the first marker point.

A tool for visualization and quantitative evaluation based on the Visualization Toolkit [28] Version 9.1.0 (Kitware, Clifton Park, New York, USA) was developed in Python. Centerline markers were manually placed on the magnitude image along the LCA, RCA, and the ascending aorta. Here, LCA refers to the combined left main and left anterior descending arteries. A volume mask with a fixed radius around the CA was refined through magnitude thresholding. The ascending aorta was segmented based on edge filtering. The segmentation masks were then applied to the 3D velocity volumes. For the comparison of 3D‐PC‐MRI and 4D flow in the phantom, identical centerline marker positions were used for both measurements. Maximum intensity projections of the segmented volumes were then visualized in a custom 3D interactive viewer. The visibility of each vessel can be set individually, which is useful in the case of visually overlapping vessels. For quantification, 100 slices were extracted perpendicularly to and along their centerlines. For each slice, the maximum velocity as a function of its position along the centerline was plotted. The postprocessing workflow is illustrated in Figure 3.

Due to limited resolution, only the proximal sections up to 25 mm along the vessel centerline were considered for quantitative flow velocity evaluation in vivo. If the first marker point is placed too close to the aorta, flow velocities for the first few slices may be severely overestimated. To account for this, first, the mean velocity $v_{\text{mean}}$ and standard deviation σ between 10 and 25 mm along the original centerline were calculated. Subsequently, an iteration over slice indices n was performed, where the first few slices are eliminated until the criterium was satisfied. In a last step, the starting point of the vessel was redefined to coincide with the location of the first accepted slice.

$$v_{n+i}\leq v_{\text{mean}}+\sigma ,i=0,1,2\;\text{for}\;n\geq 8.$$

For measurements in healthy volunteers, paired two‐tailed *t*‐tests were performed to analyze the statistical significance of differences in observed flow velocities when comparing the LCA and RCA as well as for scan‐rescan comparisons of the individual arteries.

### 2.6 CT‐FFR Evaluation

In patients, additional CT‐FFR evaluations were performed using a research application (CT cFFR Frontier version 3.5.3, Siemens Healthineers). The semiautomated software integrated a three‐dimensional coronary artery reconstruction with computational fluid dynamics, patient‐specific parameters, and a machine learning algorithm to model blood flow under resting and hyperemic conditions [29]. Vessel centerlines and boundaries were initially auto‐generated, followed by manual refinement and stenosis identification as needed. Measurements were obtained 20 mm downstream from the stenoses, with values of 0.75 or below indicating abnormal hemodynamics.

CT‐FFR results were stored as pairs of CT‐FFR values and world coordinates of the location of measurement, listed in order of position along the vessel. Subsequently, the cumulative Euclidean distances between the coordinates of measurement locations were computed to obtain the corresponding distances along the vessel centerlines to facilitate comparison of CT‐FFR to 3D‐PC‐MRI results.

## 3 Results

### 3.1 Phantom

**FIGURE 4:**
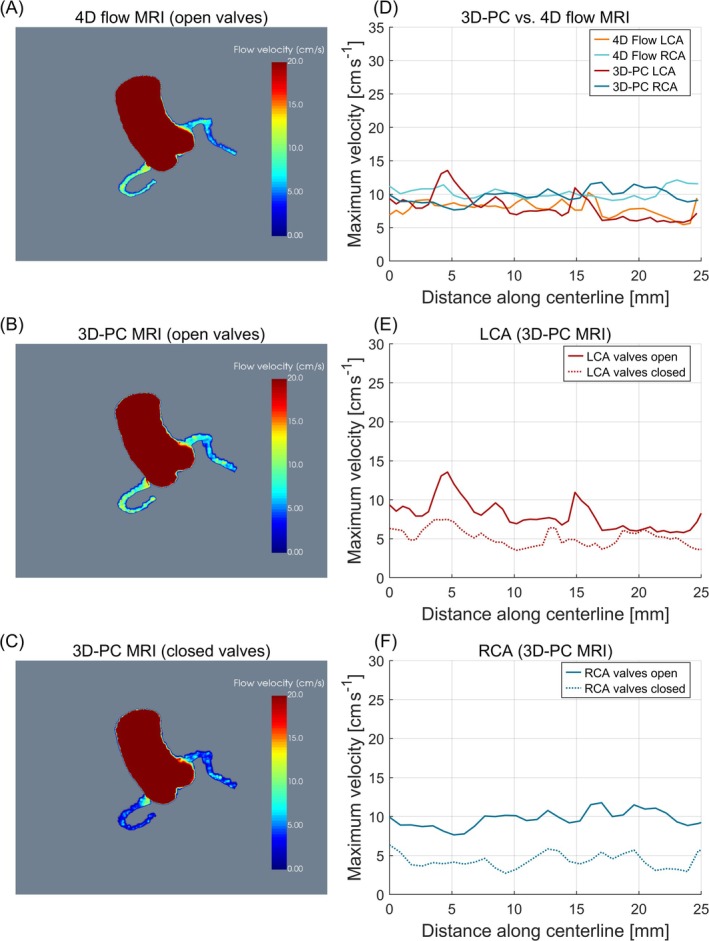
Sequence validation in the flow phantom. Velocity maximum intensity projections (MIPs) acquired with the 4D flow sequence (A) and the proposed 3D phase contrast (PC) sequence (B) for open coronary outlet valves. (C) Velocity MIPs obtained with the proposed 3D‐PC sequence for closed coronary outlet valves. (D) Quantitative evaluation of maximum flow velocities per slice along the vessel centerlines, comparing 4D flow and the proposed 3D‐PC sequence. (E) Hemodynamic comparisons of open and closed coronary outlet valves of the vessel model for the left coronary artery (LCA). (F) Hemodynamic comparisons of open and closed coronary outlet valves of the vessel model for the right coronary artery (RCA).

Phantom results are shown in Figure 4. Visual comparison of maximum intensity projections of the segmented velocity volumes acquired with 4D Flow (Figure 4A) and the proposed 3D‐PC technique (Figure 4B) demonstrates excellent agreement. This is confirmed by the quantitative evaluation in Figure 4D, showing mean point‐wise deviations of −0.13 ± 1.81 cm s^−1^ and 0.51 ± 1.55 cm s^−1^ for the proximal LCA and RCA respectively, corresponding to deviations in mean values of −1.7% and 5.0%. A paired two‐tailed *t*‐test indicated no statistically significant differences between the two techniques (p=0.62 and 0.08 for the LCA and RCA respectively).

A maximum intensity projection of the segmented velocity volume acquired using the proposed 3D‐PC sequence after closing the coronary outlet valves of the vessel model is shown in Figure 4C. As illustrated in Figure 4E,F, the flow velocity in both CA is reduced when the coronary outlet valves are closed. The differences in flow velocity curves are statistically significant (p<0.05 for both the LCA and the RCA).

Background phase correction performed using data acquired without flow resulted in flow velocity changes of less than 1.5%, and thus, background phase correction was deemed negligible.

### 3.2 Retrospective Motion and Flow Displacement Correction

**FIGURE 5:**
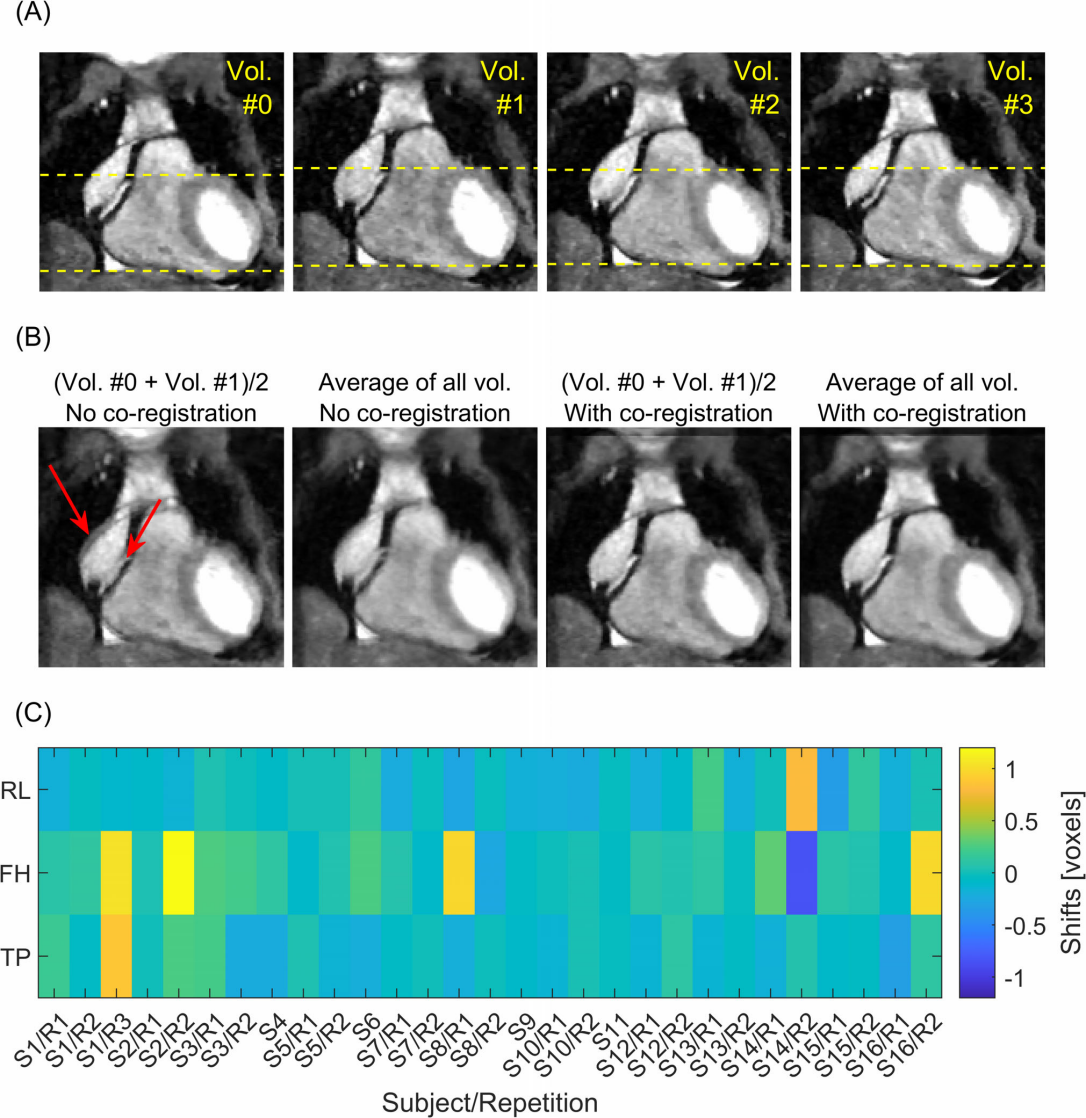
Inter‐scan motion observed for individual acquisitions of the four volumes. (A) Uncorrected cropped magnitude images for an exemplary volunteer. The yellow dashed lines bordering the myocardium demonstrate inter‐scan motion, most noticeable between volumes (Vol.) 0 and 1. (B) Different averaged images with and without image co‐registration. (C) Translational 3D shift parameters detected and subsequently corrected for all healthy volunteers.

Figure 5A,B demonstrates the necessity for inter‐scan motion correction for an exemplary volunteer measurement. A motion‐induced shift between the reference and the first acquired flow‐encoded volume is evident from both the yellow dashed lines bordering the myocardium in the FH direction in Figure 5A as well as from the average of both volumes shown in Figure 5B. Without motion correction, the average of all four volumes was blurred. After performing image registration, the alignment of the image contours, as well as the sharpness of the averaged volumes, improved. The translation parameters of volume 1 for all volunteers are plotted in Figure 5C.

The effect of both motion and spin displacement corrections on quantitative velocity profiles is illustrated for an exemplary volunteer in Figure S3.

### 3.3 Healthy Volunteers

From the 31 total acquired volunteer scans, two measurements had to be excluded from postprocessing due to poor image quality. For each remaining volunteer measurement, the respective peak and average flow velocities along the proximal artery were calculated for the LCA and RCA. Values across all volunteers were 17.6 ± 5.3 cm s^−1^ (peak, LCA), 13.3 ± 4.4 cm s^−1^ (peak, RCA), 11.9 ± 3.3 cm s^−1^ (average, LCA), and 8.4 ± 2.6 cm s^−1^ (average flow, RCA). Additionally, deviations between scans repeated in the same session were computed and averaged across all volunteers resulting in 0.3 ± 3.5 cm s^−1^ (average, LCA), −0.6 ± 2.1 cm s^−1^ (average, RCA), 2.2 ± 5.5 cm s^−1^ (peak, LCA), and −0.3 ± 4.4 cm s^−1^ (peak, RCA). Deviations between repetitions in average and peak velocities were not statistically significant for either the LCA or RCA ($p_{\mathrm{LCA},\mathrm{avg}}=0.84;p_{\mathrm{RCA},\mathrm{avg}}=0.30;p_{\mathrm{LCA},\text{peak}}=0.19;p_{\mathrm{RCA},\text{peak}}=0.75$).

**FIGURE 6:**
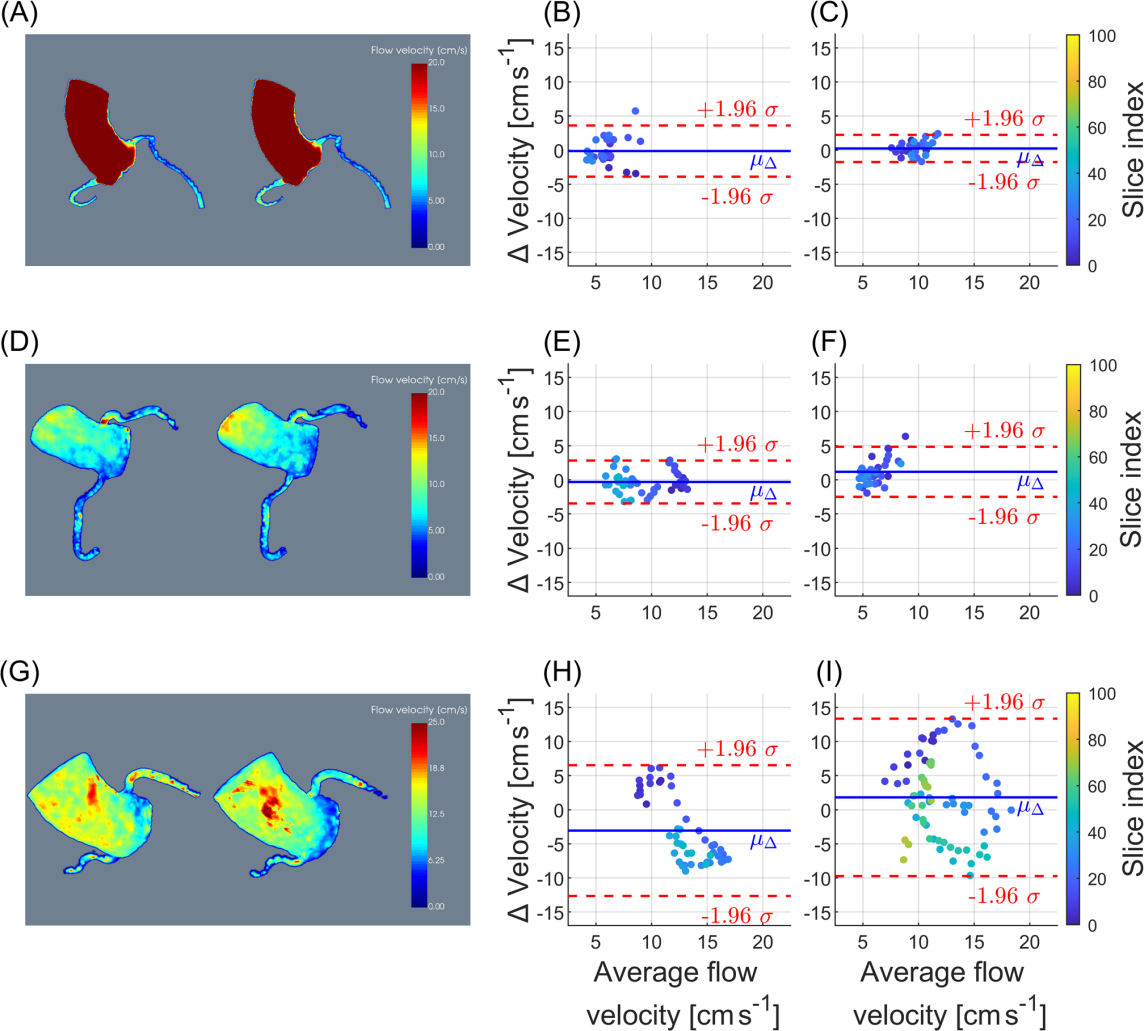
Bland–Altman analysis. Each point represents one slice along the vessel centerline, with the color‐coded slice index. (A) Maximum intensity projection images of 3D segmented velocity volumes for the initial (left) and repeated (right) scans of a realistic flow phantom. Bland–Altman diagrams comparing results for the left coronary artery (LCA) (B) and right coronary artery (RCA) (C) of the realistic flow phantom. (D) Maximum intensity projection images of 3D segmented velocity volumes for the initial (left) and repeated (right) scans of volunteer 5. Bland–Altman diagrams comparing results for the LCA (E) and RCA (F) of volunteer 5. (G) Maximum intensity projection images of 3D segmented velocity volumes for the initial (left) and repeated (right) scans of volunteer 12. Bland–Altman diagrams comparing results for the LCA (H) and RCA (I) of volunteer 12.

To further analyze scan‐rescan reproducibility, Bland–Altman analysis was performed for the peak velocities per slice along the vessel centerlines. Resulting bias, standard deviations (*σ*), limits of agreement (LOA), and mean velocities (*μ*) for all measurements are listed in Table 2. In the LCA, mean differences ranging from 0.16 to 8.10 cm s^−1^ and standard deviations ranging from 1.60 to 4.89 cm s^−1^ were observed. In the RCA, mean differences ranging from 0.12 to 5.12 cm s^−1^ and standard deviations ranging from 1.01 to 5.89 cm s^−1^ were observed. Corresponding Bland–Altman plots and 3D renderings are shown for all cases in Figures S4–S7. In all but one case (volunteer 7, RCA), absolute biases were greater in vivo compared to the flow phantom. Three exemplary cases are shown in Figure 6: Figure 6A–C corresponds to the repeated flow phantom scan, and Figure 6D–I correspond to two exemplary volunteers, rescanned within the same session. For the one volunteer who was additionally scanned during another session, intra‐ and inter‐session analyses are shown in Figure S8, showing larger limits of agreement in the inter‐session analysis. In most volunteers, the Bland–Altman plots show similar differences for neighboring slices.

**TABLE 2:** *μ*, *σ*, biases $\mu_{\Delta }$ and limits of agreement (LOA) for scan‐rescan comparisons.

Subject	$\mu_{\Delta }\pm \sigma$ LCA [cm s^−1^]	*μ* LCA [cm s^−1^]	Lower LOA LCA [cm s^−1^]	Upper LOA LCA [cm s^−1^]	$\mu_{\Delta }\pm \sigma$ RCA [cm s^−1^]	*μ* RCA [cm s^−1^]	Lower LOA RCA [cm s^−1^]	Upper LOA RCA [cm s^−1^]
Volunteer 1	0.65 ± 2.25	8.09	−3.77	5.07	−0.93 ± 2.35	7.62	−5.54	3.68
Volunteer 2	8.10 ± 2.30	15.12	3.59	12.61	5.12 ± 5.43	12.48	−5.52	15.76
Volunteer 3	0.16 ± 2.36	10.20	−4.46	4.78	1.93 ± 2.64	8.27	−3.23	7.11
Volunteer 4	0.59 ± 3.95	7.50	−7.15	8.33	0.37 ± 1.55	5.35	−2.67	3.41
Volunteer 5	−0.32 ± 1.60	9.42	−3.46	2.81	1.16 ± 1.87	6.14	−2.50	4.83
Volunteer 6	−3.33 ± 5.31	14.87	−13.74	7.08	−2.80 ± 2.05	7.48	−6.81	1.21
Volunteer 7	2.15 ± 4.68	14.75	−7.01	11.32	0.12 ± 2.38	10.06	−4.54	4.78
Volunteer 8	1.79 ± 3.24	13.70	−4.57	8.15	0.77 ± 2.34	8.06	−3.81	5.35
Volunteer 9	−1.38 ± 3.28	10.34	−7.81	5.05	−0.49 ± 3.08	7.04	−6.52	5.54
Volunteer 10	−4.08 ± 4.05	10.59	−12.03	3.86	2.05 ± 1.91	8.39	−1.70	5.81
Volunteer 11	−4.87 ± 4.34	12.75	−13.38	3.64	−1.01 ± 3.11	7.53	−7.11	5.10
Volunteer 12	−3.07 ± 4.89	12.92	−12.65	6.52	1.80 ± 5.89	12.15	−9.73	13.34
Phantom	−0.14 ± 1.91	6.04	−3.89	3.62	0.25 ± 1.01	9.72	−1.74	2.24
Volunteer 1*	0.18 ± 3.06	7.85	−5.81	6.16	−3.03 ± 2.93	6.57	−8.75	2.72

*Note*: Here *μ* symbolizes the mean across the centerline of scan‐rescan averages of maximum flow velocities. The standard deviation of scan‐rescan differences is denoted by *σ*. For the subject marked by an asterisk, the two scans were acquired on different days. In all other cases, rescans were performed within the same scan session.

### 3.4 CAD Patients

In the following, for the first three patients, reformatted CTA scans depicting the stenosis in the affected vessel are shown. A maximum intensity projection of the segmented velocity volume is displayed. The corresponding quantitative velocity curves along the vessel centerlines are plotted, and the locations of the stenoses as measured on the CTA are marked by vertical lines. Measurements performed on the fourth patient were excluded from postprocessing due to insufficient image quality.

**FIGURE 7:**
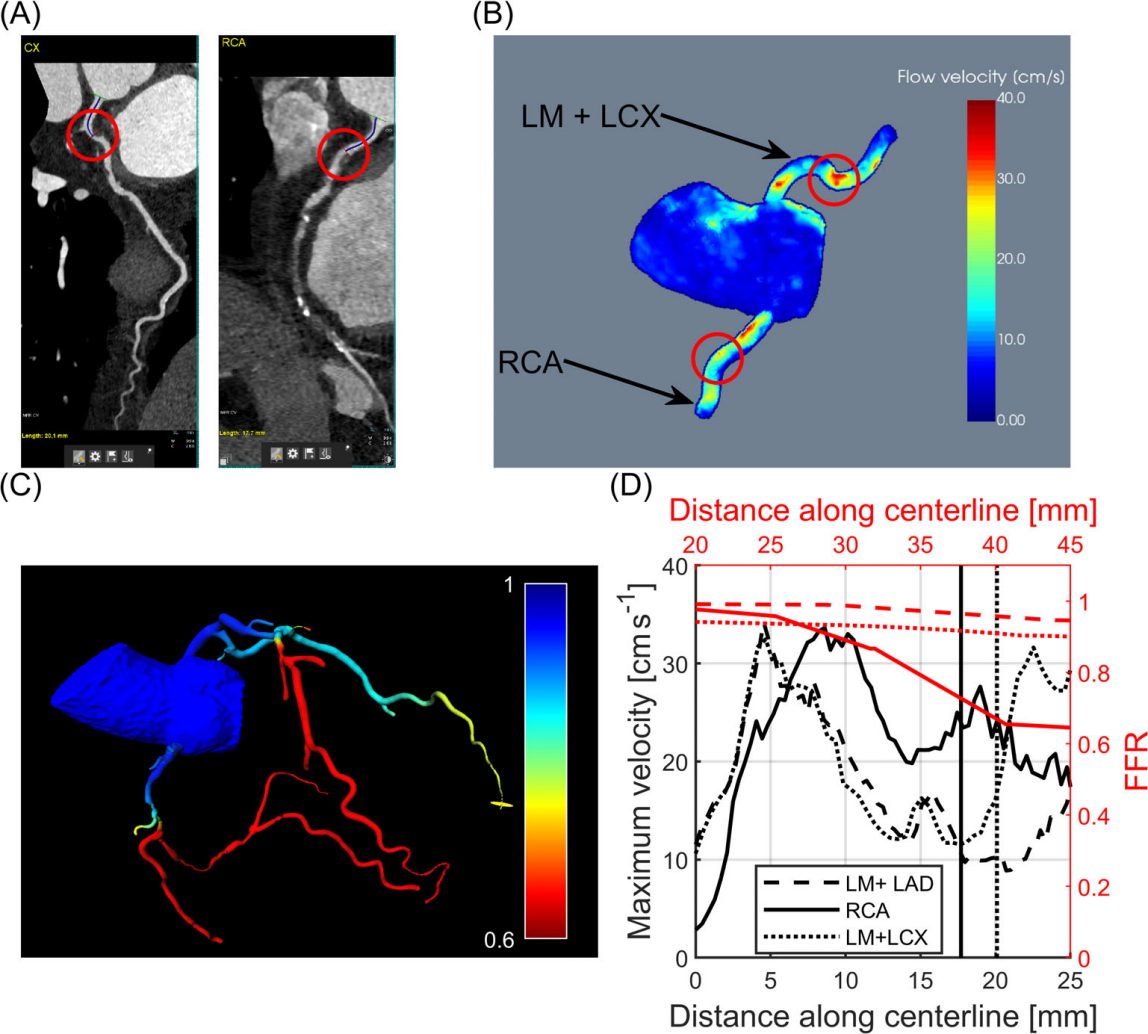
Scan results of patient 1 (male, 59 years) with stenoses in the proximal left circumflex (LCX) and right coronary artery (RCA). (A) Reformatted CT scans of the LCX and RCA. Stenoses in the proximal vessels are marked by red circles. (B) A maximum intensity projection of flow velocities acquired using our proposed 3D phase contrast (PC) sequence for the RCA and the combined left main (LM) and LCX. For display purposes, the LAD is not shown here to avoid overlap with other arteries and therefore, falsification of the apparent velocity values. (C) Distribution of fractional flow reserve (FFR) values throughout the coronary vessel tree obtained from CT scans. (D) Quantitative flow velocities corresponding to (B) as well as FFR values are plotted against distance along the vessel centerlines. The distances of the stenoses from the respective vessel ostia were measured based on the CT scans and are marked by vertical lines.

Data obtained for patient 1 (male, 59 years) with stenoses in the RCA and LCX is shown in Figure 7. In accordance with expectations, an increase in flow velocities at the level of the stenoses is observed. Regarding the RCA, the location of the stenosis also correlates with a drop in CT‐FFR. Note that it is recommended to measure the CT‐FFR values 20 mm distal to the stenosis [30]. For the LM, no noticeable change in CT‐FFR is observed.

Results for patients 2 and 3 are shown in Figures S9 and S10, respectively. Although causality must be discussed, a correlation between stenoses and increases in flow velocities is seen in all patients. For patient 2, a decrease in CT‐FFR correlates with the position of the stenosis; however, for patient 3, no noticeable change in CT‐FFR can be observed.

**FIGURE 8:**
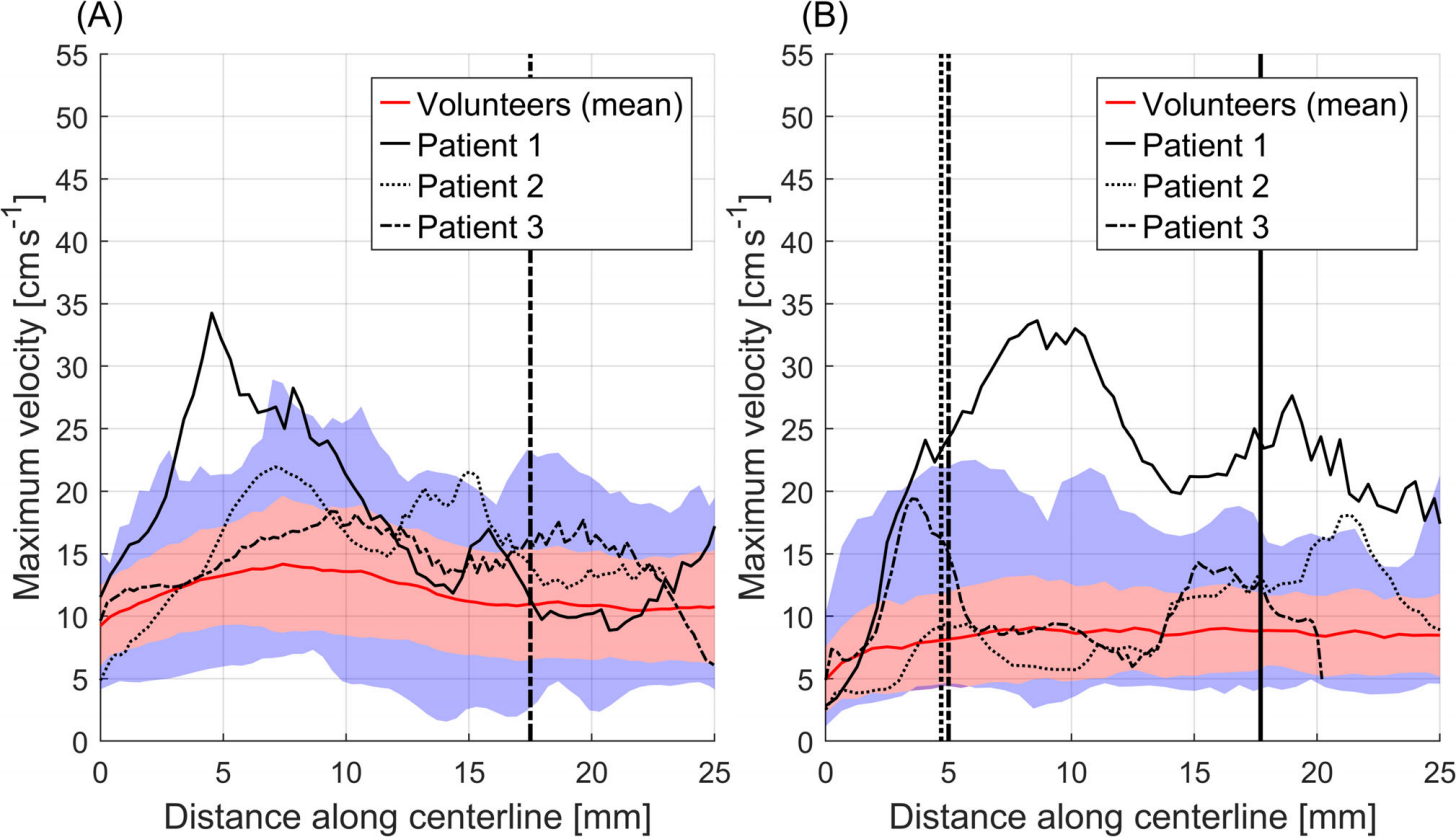
Quantitative flow curves of patients in comparison to volunteer data. (A) Comparison of flow velocities in the left coronary arteries (LCA) of patients and volunteers. (B) Comparison of flow velocities in the right coronary arteries (RCA) of patients and volunteers. In both (A) and (B), mean flow velocity curves across all volunteers and repetitions are illustrated in red. Areas shaded in red correspond to the standard deviations around the mean flow velocity curves. Areas shaded in blue correspond to the maximum and minimum flow velocities measured across all volunteers and repetitions. The locations of stenoses in the LAD and RCA are indicated by vertical lines for all patients.

Figure 8 shows quantitative velocity curves for the LCA and RCA of all patients in comparison to volunteer measurements. While the results for patients 2 and 3 lie predominantly within the range of maximum and minimum velocities observed in healthy volunteers, the flow velocities of patient 1 exceed volunteer results in large sections of the proximal RCA as well as around the first velocity peak in the LCA.

## 4 Discussion

In this work, we demonstrate the feasibility of 3D blood flow velocity measurements in the CA with MR. The proposed 3D‐PC technique was validated in a phantom, applied in healthy volunteers, and CAD patients. Inter‐scan motion correction was achieved through image co‐registration. An interactive 3D viewer was developed for flow velocity visualization and quantitative hemodynamic analysis. In a flow phantom with physiologically realistic conditions, the method showed excellent agreement with the ground truth 4D flow measurements.

Separating reference and flow encoding volume acquisitions allowed for motion correction via image registration. Furthermore, if the image quality of a single volume is unsatisfactory, it may be repeated at a lower expense regarding total scan time compared to reacquiring the entire interleaved scan. Moreover, if short waiting periods are inserted in between scans, individual scans may yield improved comfort for patients struggling to keep still for extended periods of time, as this would allow slight movements in between scans that can later be corrected.

Mean and peak flow velocities found during diastole in the proximal CA of healthy volunteers were underestimated by a factor of 2–3 compared to literature results found by Ofili et al. [14]. In their study, a Doppler angioplasty flow wire was used, which was invasively placed in the CA, potentially altering hemodynamics. Furthermore, the averaging effect of the proposed 3D‐PC MRI acquisition also leads to lower velocities as compared to a real‐time measurement performed in their study. Another important factor to consider is the comparably broad spatial resolution of (1.2 mm)^3^. The average lumen diameter of the proximal coronary arteries varies between 2.8 ± 0.5 and 4.5 ± 0.5 mm, depending on the vessel in question as well as anatomic dominance [31]. This corresponds to a diameter of 2–4 pixels, resulting in vessel blurring and underestimation of flow velocities. To account for these factors and get a fairer comparison between the results found in this work and literature values by Ofili et al., we computed the ratio of diastolic flow velocities in the proximal LAD compared to the proximal RCA for both studies. In our study, the resulting values are 1.4 and 1.3 for the average and peak flow velocities, respectively. The respective ratios calculated from the values reported by Ofili et al. were found to be 1.2 and 1.3. Hence, there is good agreement for the ratio of average flow velocity and excellent agreement for the peak flow velocity ratio.

Other previous studies investigating coronary flow velocities using 2D‐PC‐MR scans found values of 6.8 cm s^−1^ in the LAD and velocities ranging from 6.0 to 7.0 cm s^−1^ in the RCA [13, 32]. These values are slightly lower than those found in this work; however, both previous 2D‐PC‐MR studies applied temporal averaging across the entire cardiac cycle, whereas in this work, measurements were performed in a single diastolic phase. Since coronary velocities are highest in diastole [14, 21], temporally averaged values are expected to be lower.

Inter‐scan biases, standard deviations, and LOAs (Table 2) have a large spread across volunteers. Nevertheless, the pattern of neighboring slices showing similar deviations in the Bland–Altman plots shows that these deviations are correlated with the slice index. Since scan parameters were kept identical across both repetitions, we hypothesize that these patterns are mainly caused by physiological variations, such as changes in heart rate, blood pressure, and breathing [33, 34]. This hypothesis is supported by the absence of these patterns in phantom experiments, leading to smaller absolute biases compared to in vivo measurements. In general, the confidence in flow quantification is expected to decrease when moving away from the ostium to higher slice indices. This is due to decreasing vessel diameter, reduced velocity‐to‐noise ratio, and potentially increased cardiac and respiratory motion when moving distally. This effect is seen in the volunteer results, as patterns are mainly seen in the first slice indices. Remaining differences, seen both in vivo and in the phantom, are attributed to limited velocity‐to‐noise ratios and the manual post‐processing steps, such as the placement of the centerline markers and the thresholding for segmentation. Therefore, visual qualitative comparisons of repeated maximum intensity projected 3D velocity volumes are helpful in addition to quantitative Bland–Altman analyses.

Quantitative velocity curves along the vessel centerlines of patient 1 show multiple peaks. The first increase in flow velocity occurs at the ostium, since vessel diameters, wall shear stress, and pressure conditions change upon transitioning from the aorta into the coronary artery. In the LCA, a second peak appears immediately downstream of the bifurcation, where the LM branches into the LCX and LAD. This might be explained by disorganized flow effects occurring at vessel bifurcations, as demonstrated by Politis et al. through simulations for various geometries and input velocities of composite arterial coronary grafts [35]. The second peak in the RCA, as well as the third peak in the LM + LCX, cannot be explained by either effect; however, the velocity increases coincide with the locations of stenoses. This observation is in accordance with expectations based on the Bernoulli principle.

For patients 2 and 3, the cause of an increase in flow velocity at the locations of the stenoses remains ambiguous, as they are located close to the vessel ostia or bifurcations. As these increases in flow velocities at vessel bifurcations and ostia have been observed in healthy volunteers, an attribution to a CA stenosis is questionable. Hence, further patient measurements for stenoses in different locations are warranted to confirm whether the observed correlations are reproducible in other cases, which might indicate causality.

Although some discrepancies between CT‐FFR and 3D‐PC‐MRI are observed, this does not necessarily reflect the accuracy of the proposed method. While CT‐FFR has been confirmed to provide valuable insights on hemodynamics in the coronary vessels, it does have limitations that have been discussed in other works, for example by Rajiah et al. [30]. Limitations include errors in vessel segmentation that lead to wrong assumptions for lumen diameters and, subsequently, errors in FFR estimates. Furthermore, CT‐FFR is calculated from simulated hyperemia, which may differ from actual hyperemia, and thus may yield misleading results. Also, sex differences in CT‐FFR values have been reported for the same degree of stenoses [36]. Hence, CT‐FFR values should not be interpreted in isolation but always in combination with other factors. Regarding this study, CT‐FFR therefore should not be considered to represent the ground truth, but rather an alternative method for analyzing hemodynamics which, just as the proposed 3D‐PC‐MRI approach, has some limitations.

### 4.1 Limitations

A major limitation of this study remains the duration of the 3D‐PC acquisition. The average time required for obtaining all four datasets during free breathing across all subjects and repetitions was (16:07 ± 4:47) min. One possible approach to improve data efficiency is the use of respiratory motion binning [37]. Another limitation of the method is the limited image resolution. Improved resolution would be desirable due to the small vessel sizes; however, it comes at the expense of prolonged scan times and reduced signal‐to‐noise ratios. One approach to achieve higher resolution while maintaining reasonable scan times, used in a study by Blanken et al., is a targeted acquisition, restricting the acquisition to one CA [2]. In our study, however, the benefit of a 3D whole‐heart acquisition was chosen, simplifying slice planning and yielding anatomic information of the entire heart in addition to flow information for the proximal coronary arteries.

Another limitation is that the hemodynamic significance of stenoses becomes most apparent during stress; however, the long scan times and acquisition window durations, as well as the need for a steady heart rate, do not allow for the performance of 3D‐PC‐MRI during exercise or pharmacologically induced stress.

During post‐processing, potential error sources include the manual placement of centerline markers, interpolation, and vessel segmentation. Each of these factors may cause deviations between the segmentation mask and the actual outline of the CA, consequently influencing the quantitative flow evaluation. Although care was taken to minimize this impact by placing the first point at the level of the ostium, this effect can only be mitigated by using a fully automatic segmentation and quantification pipeline, warranted for future work. Assuming that flow velocities are highest in the center of the vessel cross‐section, maximum flow velocities per cross‐sectional slice are less sensitive to the boundaries of the segmentation mask than the respective mean flow velocities. Hence, it was chosen to investigate maximum flow velocities per slice along the vessel centerlines.

Furthermore, a lack of ground truth for the hemodynamics in the CA remains a limitation, preventing a comparison of our acquired results to the underlying ground truth. Moreover, the proposed method was only tested in a small patient cohort, and the cause of the observed increases in flow velocities remains ambiguous. Although enlarging the patient cohort would be desirable, the present patient cohort serves as a proof‐of‐principle.

Another limitation of our method is the non‐applicability in patients who previously received coronary stents since susceptibility artifacts severely degrade image quality. We further expect poor performance in patients with arrhythmia as changes in heart rate would lead to a mismatch between the acquisition window and the quiescent cardiac phase. Changes in the acquired cardiac phase may even be caused by slight physiologic variations in between scans, since the trigger delay is not readjusted individually for each scan.

## 5 Conclusion

In conclusion, a method for non‐invasive 3D blood flow velocity imaging in the CA at 3 T is proposed and tested in healthy volunteers as well as CAD patients. Increases in flow velocities correlate with the locations of stenoses; however, some ambiguity regarding the root cause of the observed velocity variations remains. Nevertheless, the preliminary results presented in this work seem promising and warrant further investigation in a larger patient cohort in future works. The ability to directly assess flow velocities in the CA non‐invasively and simultaneously acquire anatomical images could offer new opportunities for diagnosing and monitoring the progression of coronary artery disease, benefiting both patients and clinicians.

## Conflicts of Interest

D.L. receives a PhD stipend from Siemens Healthineers. M.S., J.W., P.S. and D.G. are employees of Siemens Healthineers.

## Supporting information


**Figure S1:** Simplified flow phantom setup. The phantom consists of a tube with a cross‐sectional area of 200 mm^2^ (red), flow was assumed laminar at the measured ROI. Flow rates were varied using the pump; an ultrasound flow‐meter (FD‐H20, KEYENCE DEUTSCHLAND GmbH, Neu‐Isenburg, Germany) was used as a reference measure. Flow directions in the tube are indicated by arrows. The approximate position of the FOV during measurement is illustrated by the black rectangle.
**Figure S2:** Maximum flow velocities measured with the proposed 3D‐PC technique compared to velocities derived from flowmeter for different flow rates. Maximum flow velocities per cross‐sectional slice were averaged across the tube section. Excellent correlation is observed, the offset is due to the averaging measurement performed by the flowmeter in contrast to the maximum velocity measured by 3D‐PC‐MRI.
**Figure S3:** Quantitative influence of correction methods. (A) Quantitative velocity curves for the left coronary artery (LCA) of an exemplary volunteer. (B) Quantitative velocity curves for the right coronary artery (RCA) of an exemplary volunteer. In both a and b uncorrected flow velocity curves (red) are shown in comparison to velocity curves after motion correction (blue) and following both motion and displacement correction (orange).
**Figure S4:** Intra‐session Bland–Altman analysis for volunteers 1–3. Each point represents one slice along the vessel centerline, with the color‐coded slice index. (A) Maximum intensity projection images of 3D segmented velocity volumes for the initial (left) and repeated (right) scans of volunteer 1. Bland–Altman diagrams comparing results for the left coronary artery (LCA) (B) and right coronary artery (RCA) (C) of volunteer 1. (D) Maximum intensity projection images of 3D segmented velocity volumes for the initial (left) and repeated (right) scans of volunteer 2. Bland–Altman‐diagrams comparing results for the LCA (E) and RCA (F) of volunteer 2. (G) Maximum intensity projection images of 3D segmented velocity volumes for the initial (left) and repeated (right) scans of volunteer 3. Bland–Altman‐diagrams comparing results for the LCA (H) and RCA (I) of volunteer 3.
**Figure S5:** Intra‐session Bland–Altman analysis for volunteers 4–6. Each point represents one slice along the vessel centerline, with the color‐coded slice index. (A) Maximum intensity projection images of 3D segmented velocity volumes for the initial (left) and repeated (right) scans of volunteer 4. Bland–Altman diagrams comparing results for the left coronary artery (LCA) (B) and right coronary artery (RCA) (C) of volunteer 4. (D) Maximum intensity projection images of 3D segmented velocity volumes for the initial (left) and repeated (right) scans of volunteer 5. Bland–Altman diagrams comparing results for the LCA (E) and RCA (F) of volunteer 5. (G) Maximum intensity projection images of 3D segmented velocity volumes for the initial (left) and repeated (right) scans of volunteer 6. Bland–Altman diagrams comparing results for the LCA (H) and RCA (I) of volunteer 6.
**Figure S6:** Intra‐session Bland–Altman analysis for volunteers 7–9. Each point represents one slice along the vessel centerline, with the color‐coded slice index. (A) Maximum intensity projection images of 3D segmented velocity volumes for the initial (left) and repeated (right) scans of volunteer 7. Bland–Altman diagrams comparing results for the left coronary artery (LCA) (B) and right coronary artery (RCA) (C) of volunteer 7. (D) Maximum intensity projection images of 3D segmented velocity volumes for the initial (left) and repeated (right) scans of volunteer 8. Bland–Altman diagrams comparing results for the LCA (E) and RCA (F) of volunteer 8. (G) Maximum intensity projection images of 3D segmented velocity volumes for the initial (left) and repeated (right) scans of volunteer 9. Bland–Altman diagrams comparing results for the LCA (H) and RCA (I) of volunteer 9.
**Figure S7:** Intra‐session Bland–Altman analysis for volunteers 10–12. Each point represents one slice along the vessel centerline, with the color‐coded slice index. (A) Maximum intensity projection images of 3D segmented velocity volumes for the initial (left) and repeated (right) scans of volunteer 10. Bland–Altman diagrams comparing results for the left coronary artery (LCA) (B) and right coronary artery (RCA) (C) of volunteer 10. (D) Maximum intensity projection images of 3D segmented velocity volumes for the initial (left) and repeated (right) scans of volunteer 11. Bland–Altman diagrams comparing results for the LCA (E) and RCA (F) of volunteer 11. (G) Maximum intensity projection images of 3D segmented velocity volumes for the initial (left) and repeated (right) scans of volunteer 12. Bland–Altman diagrams comparing results for the LCA (H) and RCA (I) of volunteer 12.
**Figure S8:** Intra‐session and inter‐session Bland–Altman analyses for volunteer 1. Each point represents one slice along the vessel centerline, with the color‐coded slice index. (A) 3D segmented velocity volumes for the initial (left) and repeated (right) scans acquired within one scan session. Intra‐session Bland–Altman diagrams comparing results for the left coronary artery (LCA) (B) and right coronary artery (RCA) (C). (D) 3D segmented velocity volumes for the initial (left) and repeated (right) scans acquired on different days. Inter‐session Bland–Altman diagrams comparing results for the LCA (E) and RCA (F).
**Figure S9:** Scan results of patient 2 (male, 74 years) with confirmed stenoses in the proximal left main (LM). (A) Reformatted Flash‐CT scans of the left coronary artery (LCA) where the proximal stenosis is marked by a red circle. (B) A maximum intensity projection of flow velocities acquired using our proposed 3D phase contrast (PC) sequence for the right coronary artery (RCA) and the combined LM and left anterior descending (LAD), where the proximal stenosis in the LM is indicated by a red circle. For display purposes, the LCX is not shown here to avoid overlap with other arteries and therefore, falsification of the apparent velocity values. (C) Distribution of fractional flow reserve (FFR) values throughout the coronary vessel tree obtained from CT scans. (D) Quantitative flow velocities corresponding to (B) as well as FFR‐values are plotted against distance along the vessel centerlines. The distances of the stenoses from the respective vessel ostia were measured based on the CT scans and are marked by vertical lines. An increase in flow velocity at the location of the stenosis is observed.
**Figure S10:** Scan results of patient 3 (female, 82 years) with triple‐vessel‐disease. A stenosis in the LM artery is located directly proximal to the bifurcation where the LM splits into the LAD and LCX. Another stenosis was confirmed in the RCA shortly distal to the vessel ostium. (A) Reformatted Flash‐CT scans of the LM and left anterior descending (LAD) and right coronary artery (RCA) where the proximal stenoses are marked by a red circle. (B) A maximum intensity projection of flow velocities acquired using our proposed 3D phase contrast (PC) sequence for the RCA and the combined LM and LAD, where the proximal stenoses in the LM and RCA are indicated by red circles. For display purposes, the LCX is not shown here to avoid overlap with other arteries and therefore, falsification of the apparent velocity values. (C) Distribution of fractional flow reserve (FFR) values throughout the coronary vessel tree obtained from CT scans. (D) Quantitative flow velocities corresponding to (B) as well as FFR‐values are plotted against distance along the vessel centerlines. The distances of the stenoses from the respective vessel ostia were measured based on the CT scans and are marked by vertical lines. Flow velocities increase at the locations of the stenoses.

## Data Availability

The patient data underlying this article cannot be shared publicly due to privacy considerations. Volunteer data analyzed during the current study are available from the corresponding author (DL) upon reasonable request.
