# Supplementary material for: Coronary Artery Blood Flow Imaging Using 3D Flow MRI
Source: Magn Reson Med. 2025 Oct 22;95(3):1545–59. doi: 10.1002/mrm.70147 (PMC12746356; doi:10.1002/mrm.70147)
Supplement: Supplementary file 1 — Figure S1: Simplified flow phantom setup. The phantom consists of a tube with a cross‐sectional area of 200 mm2 (red), flow was assumed laminar at the measured ROI. Flow rates were varied using the pump; an ultrasound flow‐meter (FD‐H20, KEYENCE DEUTSCHLAND GmbH, Neu‐Isenburg, Germany) was used as a reference measure. Flow directions in the tube are indicated by arrows. The approximate position of the FOV during measurement is illustrated by the black rectangle. Figure S2: Maximum flow velocities measured with the proposed 3D‐PC technique compared to velocities derived from flowmeter for different flow rates. Maximum flow velocities per cross‐sectional slice were averaged across the tube section. Excellent correlation is observed, the offset is due to the averaging measurement performed by the flowmeter in contrast to the maximum velocity measured by 3D‐PC‐MRI. Figure S3: Quantitative influence of correction methods. (A) Quantitative velocity curves for the left coronary artery (LCA) of an exemplary volunteer. (B) Quantitative velocity curves for the right coronary artery (RCA) of an exemplary volunteer. In both a and b uncorrected flow velocity curves (red) are shown in comparison to velocity curves after motion correction (blue) and following both motion and displacement correction (orange). Figure S4: Intra‐session Bland–Altman analysis for volunteers 1–3. Each point represents one slice along the vessel centerline, with the color‐coded slice index. (A) Maximum intensity projection images of 3D segmented velocity volumes for the initial (left) and repeated (right) scans of volunteer 1. Bland–Altman diagrams comparing results for the left coronary artery (LCA) (B) and right coronary artery (RCA) (C) of volunteer 1. (D) Maximum intensity projection images of 3D segmented velocity volumes for the initial (left) and repeated (right) scans of volunteer 2. Bland–Altman‐diagrams comparing results for the LCA (E) and RCA (F) of volunteer 2. (G) Maximum intensity projecti [file MRM-95-1545-s001.docx]

**Coronary artery blood flow imaging using 3D phase contrast MRI**

**Supplementary Information**


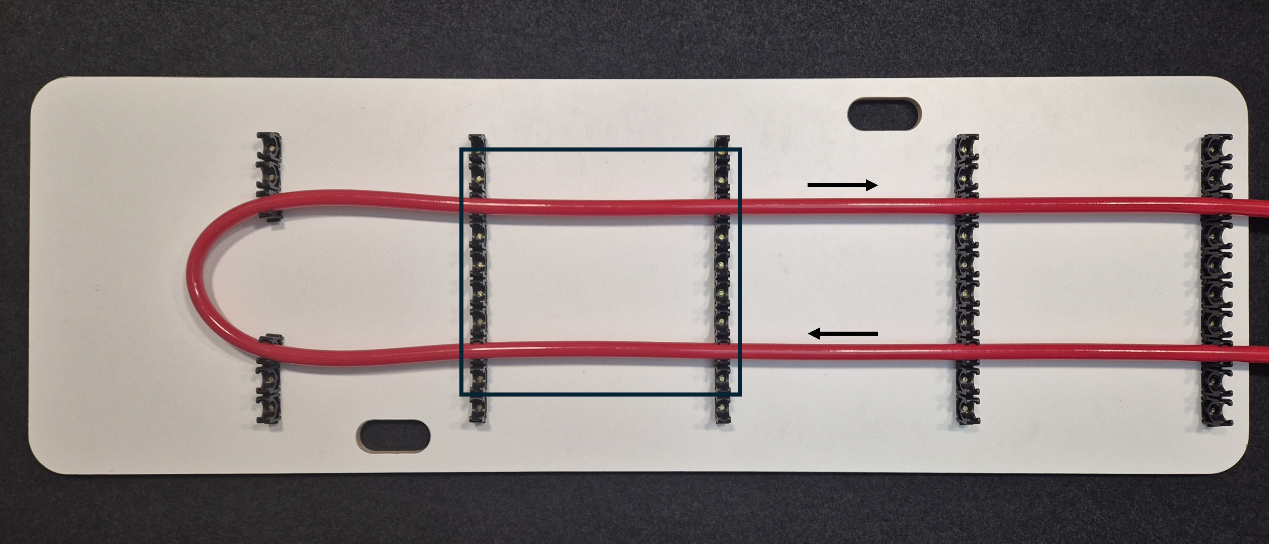


**Supplementary Figure S1:** **Simplified flow phantom setup.** *The phantom consists of a tube with a cross-sectional area of 200mm^2^ (red), flow was assumed laminar at the measured ROI. Flow rates were varied using the pump; an ultrasound flow-meter (FD-H20, KEYENCE DEUTSCHLAND GmbH, Neu-Isenburg, Germany) was used as a reference measure. Flow directions in the tube are indicated by arrows. The approximate position of the FOV during measurement is illustrated by the black rectangle.*


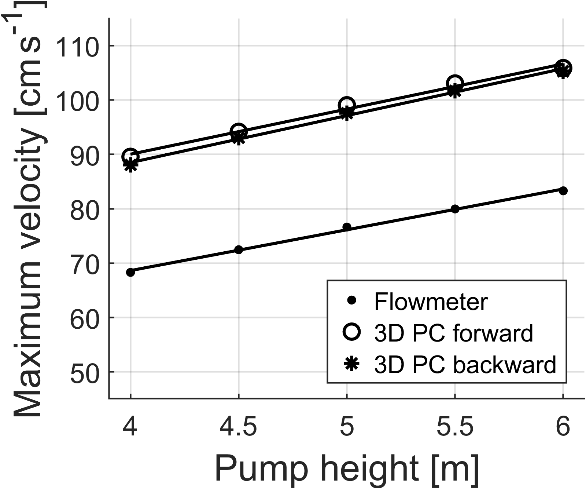


**Supplementary Figure S2: Maximum** **flow velocities measured with the proposed 3D-PC technique compared to velocities derived from flowmeter for different flow rates.** *Maximum flow velocities per cross-sectional slice were averaged across the tube section. Excellent correlation is observed, the offset is due to the averaging measurement performed by the flowmeter in contrast to the maximum velocity measured by 3D-PC-MRI.*


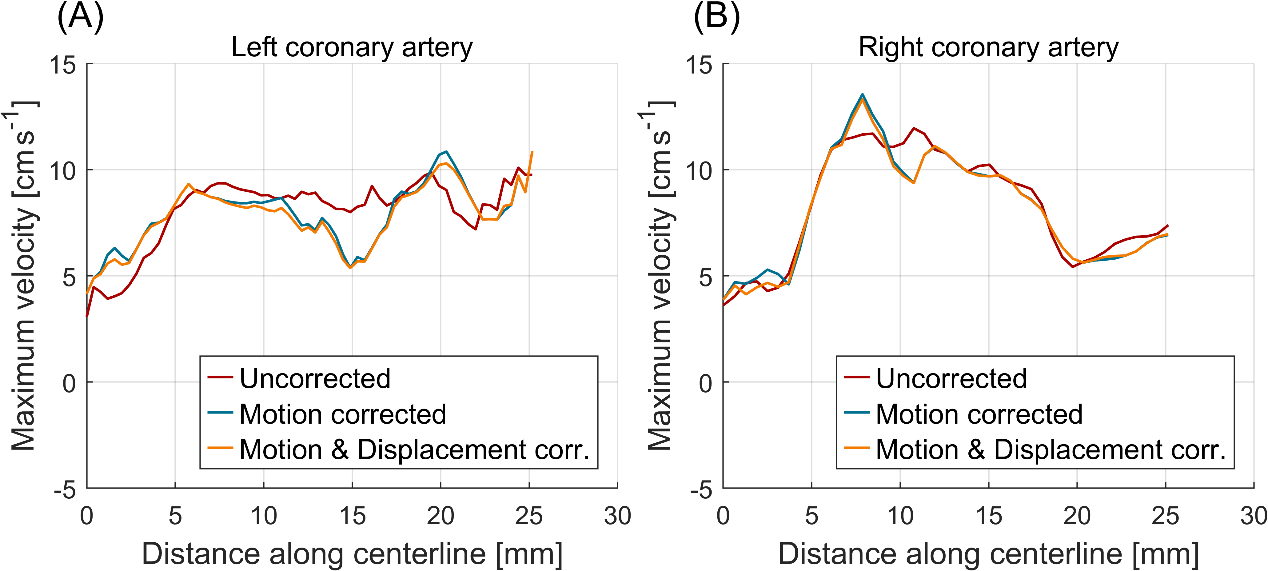


**Supplementary Figure S3:** **Quantitative influence of correction methods.** *(A) Quantitative velocity curves for the left coronary artery (LCA) of an exemplary volunteer. (B) Quantitative velocity curves for the right coronary artery (RCA) of an exemplary volunteer. In both a and b uncorrected flow velocity curves (red) are shown in comparison to velocity curves after motion correction (blue) and following both motion and displacement correction (orange).*


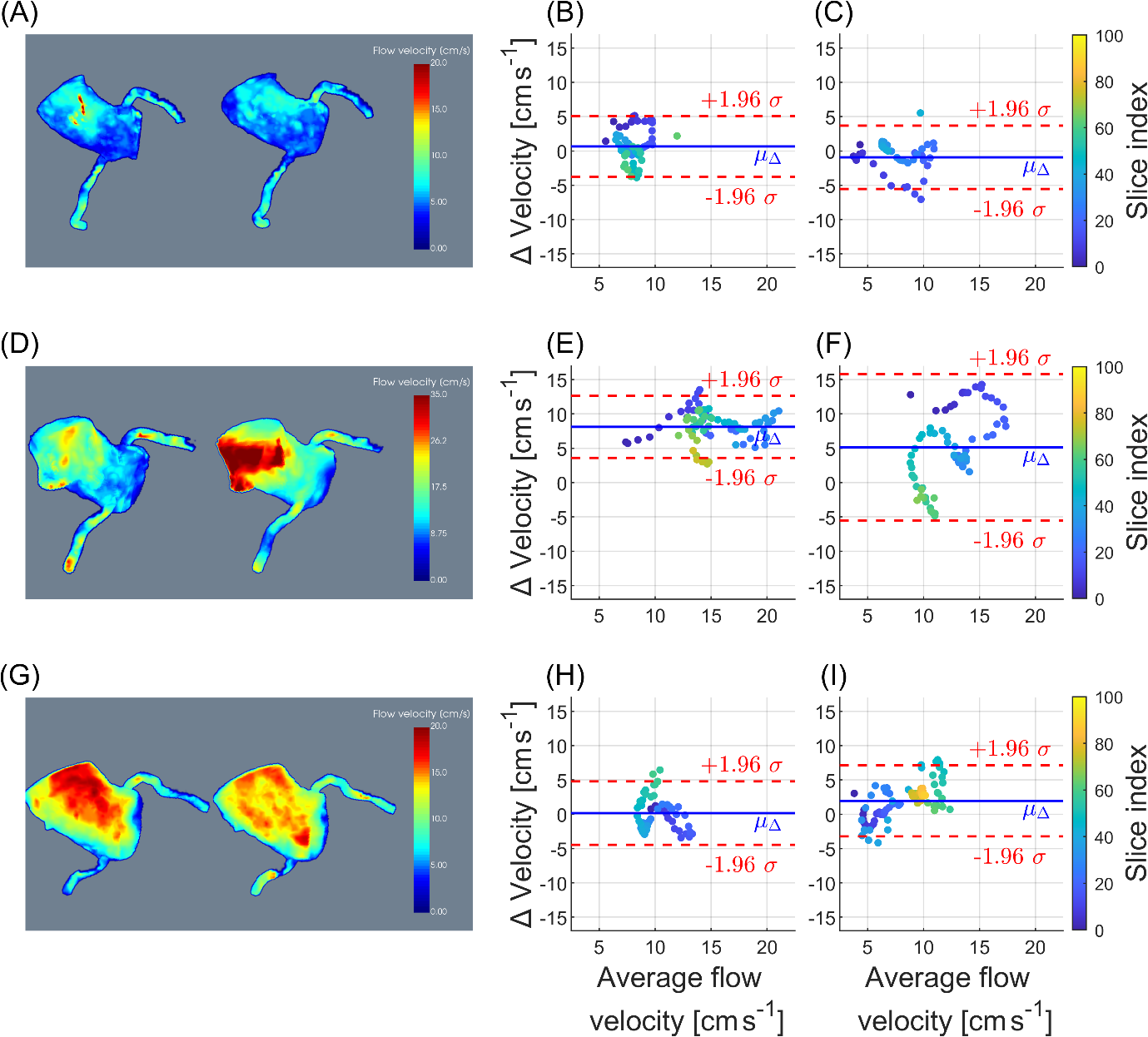


**Supplementary Figure S4:** **Intra-session** **Bland-Altman analysis for volunteers 1-3.** *Each point represents one slice along the vessel centerline, with the color-coded slice index. (A) Maximum intensity projection images of 3D segmented velocity volumes for the initial (left) and repeated (right) scans of volunteer 1. Bland-Altman diagrams comparing results for the left coronary artery (LCA) (B) and right coronary artery (RCA) (C) of volunteer 1. (D) Maximum intensity projection images of 3D segmented velocity volumes for the initial (left) and repeated (right) scans of volunteer 2. Bland-Altman-diagrams comparing results for the LCA (E) and RCA (F) of volunteer 2. (G) Maximum intensity projection images of 3D segmented velocity volumes for the initial (left) and repeated (right) scans of volunteer 3. Bland-Altman-diagrams comparing results for the LCA (H) and RCA (I) of volunteer 3.*


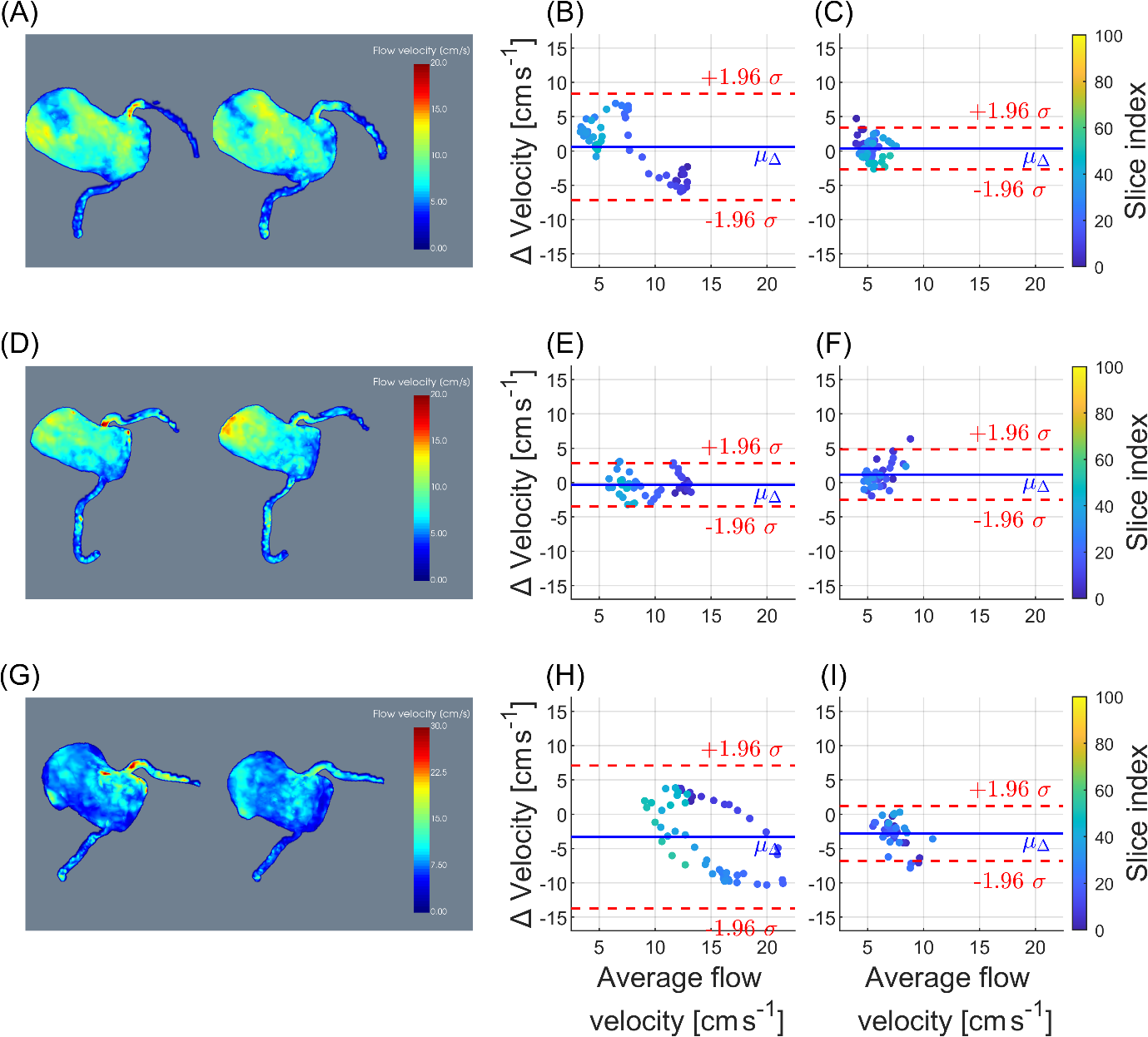


**Supplementary Figure S5:** **Intra-session** **Bland-Altman analysis for volunteers 4-6.** *Each point represents one slice along the vessel centerline, with the color-coded slice index. (A) Maximum intensity projection images of 3D segmented velocity volumes for the initial (left) and repeated (right) scans of volunteer 4. Bland-Altman diagrams comparing results for the left coronary artery (LCA) (B) and right coronary artery (RCA) (C) of volunteer 4. (D) Maximum intensity projection images of 3D segmented velocity volumes for the initial (left) and repeated (right) scans of volunteer 5. Bland-Altman diagrams comparing results for the LCA (E) and RCA (F) of volunteer 5. (G) Maximum intensity projection images of 3D segmented velocity volumes for the initial (left) and repeated (right) scans of volunteer 6. Bland-Altman diagrams comparing results for the LCA (H) and RCA (I) of volunteer 6.*


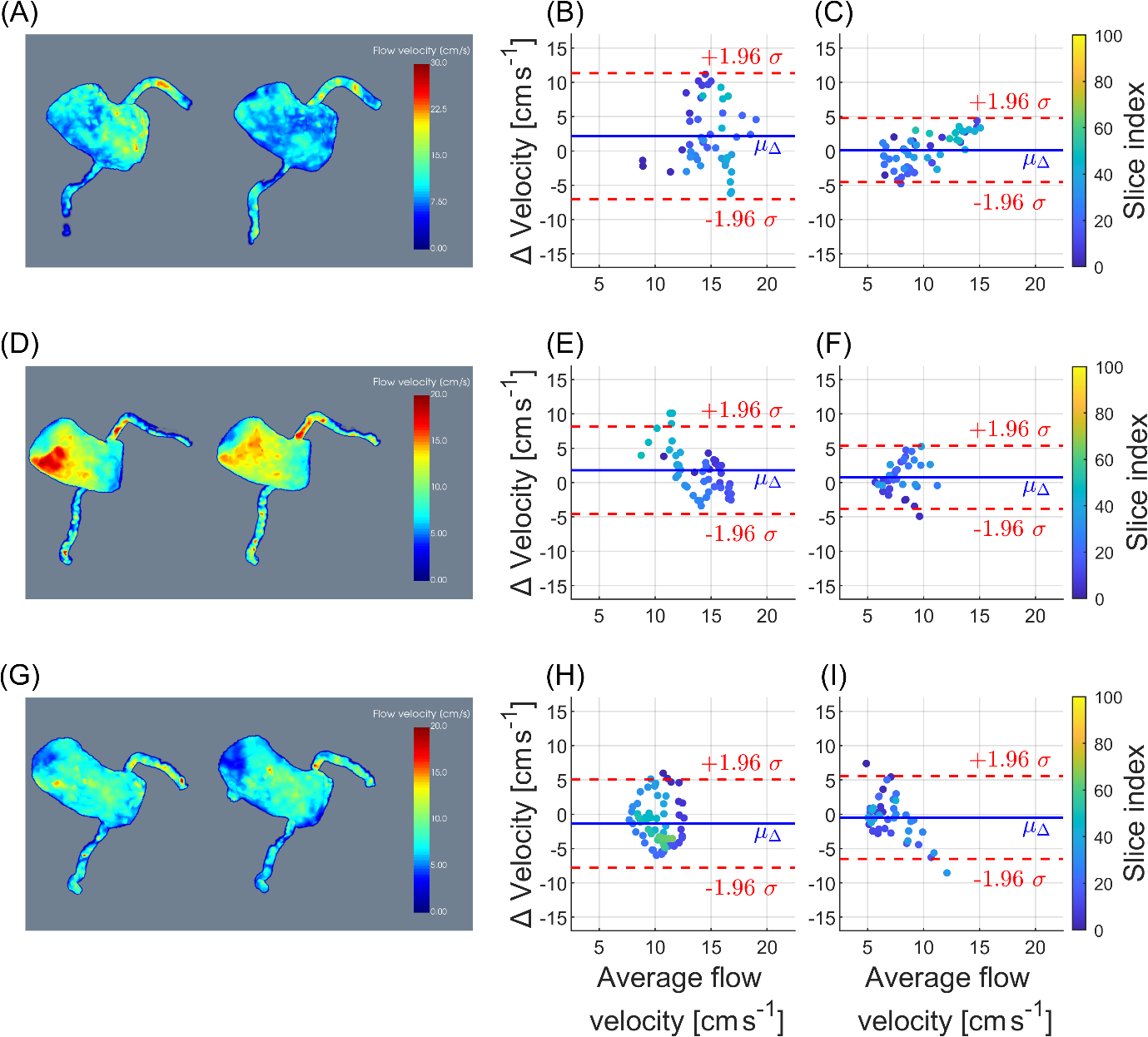


**Supplementary Figure S6:** **Intra-session** **Bland-Altman analysis for volunteers 7-9.** *Each point represents one slice along the vessel centerline, with the color-coded slice index. (A) Maximum intensity projection images of 3D segmented velocity volumes for the initial (left) and repeated (right) scans of volunteer 7. Bland-Altman diagrams comparing results for the left coronary artery (LCA) (B) and right coronary artery (RCA) (C) of volunteer 7. (D) Maximum intensity projection images of 3D segmented velocity volumes for the initial (left) and repeated (right) scans of volunteer 8. Bland-Altman diagrams comparing results for the LCA (E) and RCA (F) of volunteer 8. (G) Maximum intensity projection images of 3D segmented velocity volumes for the initial (left) and repeated (right) scans of volunteer 9. Bland-Altman diagrams comparing results for the LCA (H) and RCA (I) of volunteer 9.*


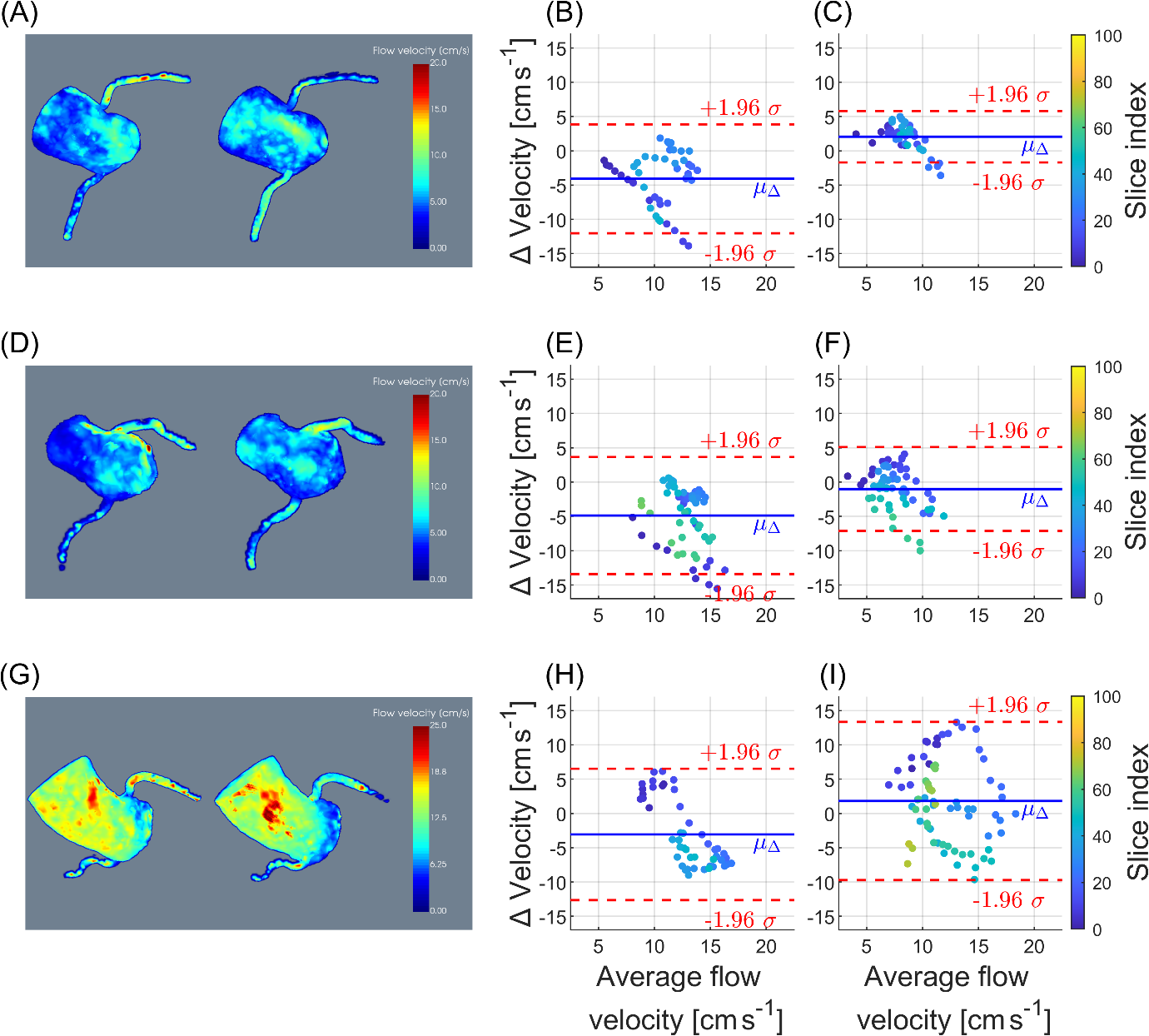


**Supplementary Figure S7:** **Intra-session** **Bland-Altman analysis for volunteers 10-12.** *Each point represents one slice along the vessel centerline, with the color-coded slice index. (A) Maximum intensity projection images of 3D segmented velocity volumes for the initial (left) and repeated (right) scans of volunteer 10. Bland-Altman diagrams comparing results for the left coronary artery (LCA) (B) and right coronary artery (RCA) (C) of volunteer 10. (D) Maximum intensity projection images of 3D segmented velocity volumes for the initial (left) and repeated (right) scans of volunteer 11. Bland-Altman diagrams comparing results for the LCA (E) and RCA (F) of volunteer 11. (G) Maximum intensity projection images of 3D segmented velocity volumes for the initial (left) and repeated (right) scans of volunteer 12. Bland-Altman diagrams comparing results for the LCA (H) and RCA (I) of volunteer 12.*


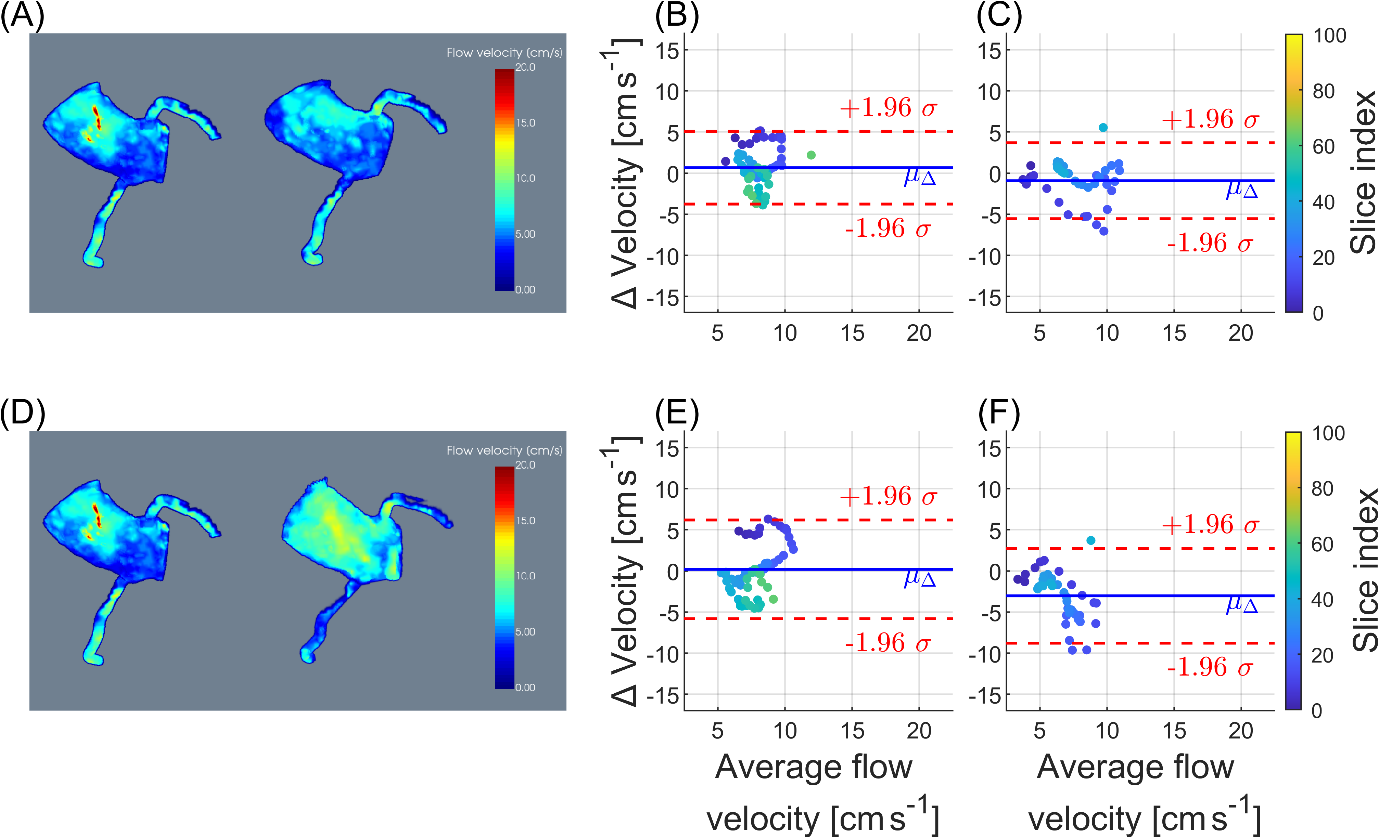


**Supplementary Figure S8:** **Intra-session** **and inter-session** **Bland-Altman analyses for volunteer 1.** *Each point represents one slice along the vessel centerline, with the color-coded slice index. (A) Maximum intensity projection images of 3D segmented velocity volumes for the initial (left) and repeated (right) scans acquired within one scan session. Intra-session Bland-Altman diagrams comparing results for the left coronary artery (LCA) (B) and right coronary artery (RCA) (C). (D) Maximum intensity projection images of 3D segmented velocity volumes for the initial (left) and repeated (right) scans acquired on different days. Inter-session Bland-Altman diagrams comparing results for the LCA (E) and RCA (F).*


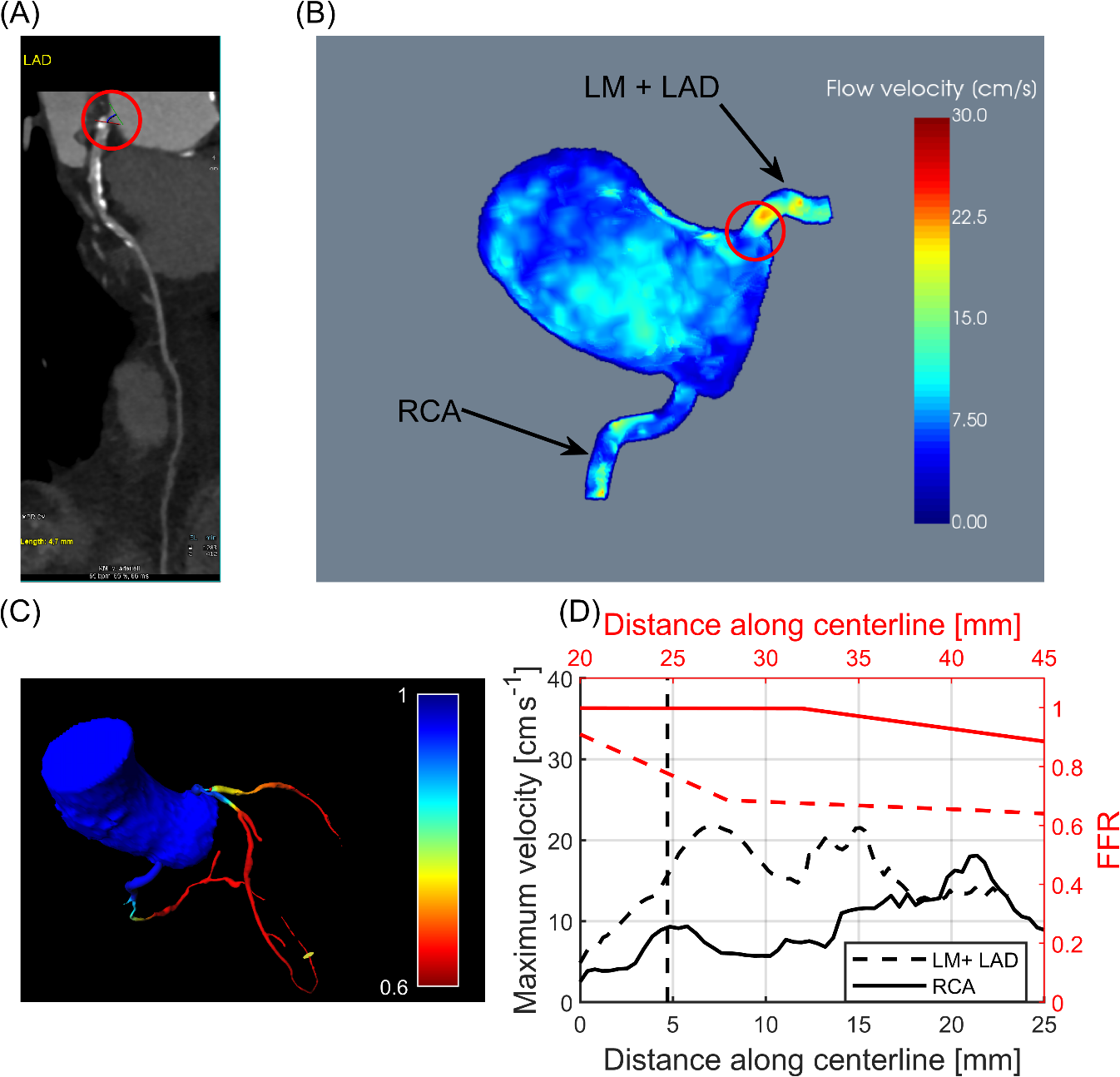


**Supplementary Figure S9:** **Scan results of patient 2 (male, 74 years) with confirmed stenoses in the proximal left main (LM).** *(A) Reformatted Flash-CT scans of the left coronary artery (LCA) where the proximal stenosis is marked by a red circle. (B) A maximum intensity projection of flow velocities acquired using our proposed 3D phase contrast (PC) sequence for the right coronary artery (RCA) and the combined LM and left anterior descending (LAD), where the proximal stenosis in the LM is indicated by a red circle. For display purposes, the LCX is not shown here to avoid overlap with other arteries and therefore, falsification of the apparent velocity values. (C) Distribution of fractional flow reserve (FFR) values throughout the coronary vessel tree obtained from CT scans. (D) Quantitative flow velocities corresponding to (B) as well as FFR-values are plotted against distance along the vessel centerlines. The distances of the stenoses from the respective vessel ostia were measured based on the CT scans and are marked by vertical lines. An increase in flow velocity at the location of the stenosis is observed.*

*
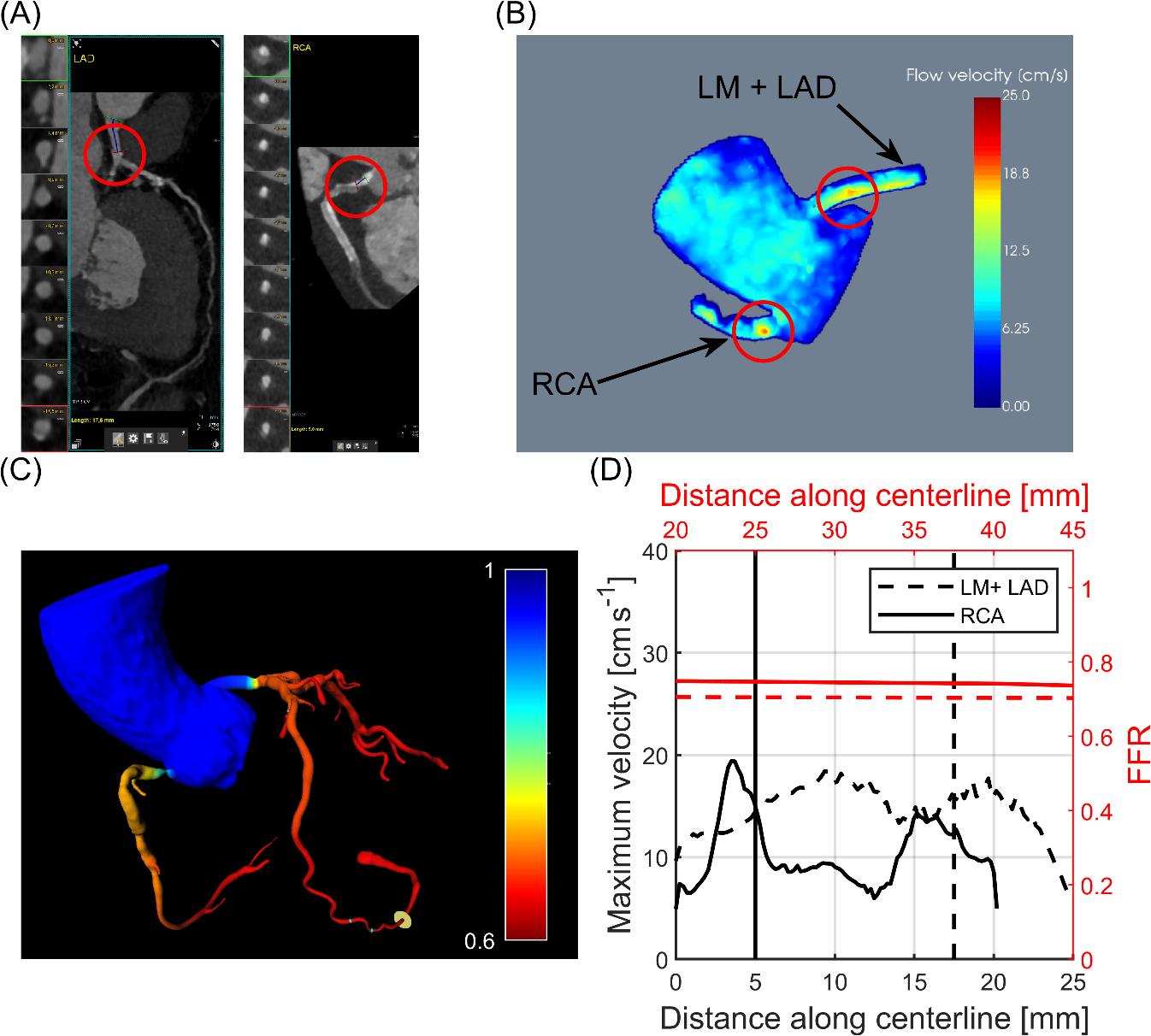
*

**Supplementary Figure S10:** **Scan results of patient 3 (female, 82 years) with triple-vessel-disease.** *A stenosis in the LM artery is located directly proximal to the bifurcation where the LM splits into the LAD and LCX. Another stenosis was confirmed in the RCA shortly distal to the vessel ostium. (A) Reformatted Flash-CT scans of the LM and left anterior descending (LAD) and right coronary artery (RCA) where the proximal stenoses are marked by a red circle. (B) A maximum intensity projection of flow velocities acquired using our proposed 3D phase contrast (PC) sequence for the RCA and the combined LM and LAD, where the proximal stenoses in the LM and RCA are indicated by red circles. For display purposes, the LCX is not shown here to avoid overlap with other arteries and therefore, falsification of the apparent velocity values. (C) Distribution of fractional flow reserve (FFR) values throughout the coronary vessel tree obtained from CT scans. (D) Quantitative flow velocities corresponding to (B) as well as FFR-values are plotted against distance along the vessel centerlines. The distances of the stenoses from the respective vessel ostia were measured based on the CT scans and are marked by vertical lines. Flow velocities increase at the locations of the stenoses.*
